# Relating characteristics of global biodiversity targets to reported progress

**DOI:** 10.1111/cobi.13322

**Published:** 2019-06-05

**Authors:** Elizabeth J. Green, Graeme M. Buchanan, Stuart H. M. Butchart, Georgina M. Chandler, Neil D. Burgess, Samantha L. L. Hill, Richard D. Gregory

**Affiliations:** ^1^ Centre for Conservation Science Royal Society for the Protection of Birds The Lodge, Potton Road Sandy SG19 2DL U.K.; ^2^ UN Environment World Conservation Monitoring Centre (UNEP‐WCMC) 219 Huntingdon Road Cambridge CB3 0DL U.K.; ^3^ BirdLife International David Attenborough Building, Pembroke Street Cambridge CB2 3QZ U.K.; ^4^ Department of Zoology University of Cambridge Downing Street Cambridge CB2 3EJ U.K.; ^5^ Centre for Macroecology, Evolution and Climate, Natural History Museum University of Copenhagen Copenhagen DK‐2100 Denmark; ^6^ Department of Life Sciences Natural History Museum Cromwell Road London SW7 5BD U.K.; ^7^ Centre for Biodiversity & Environment Research University College London London WC1H 0AG U.K.

**Keywords:** Aichi Biodiversity Targets, Convention on Biological Diversity, expert assessment, post‐2020, SMART framework, Strategic Plan for Biodiversity, Convenio sobre la Diversidad Biológica, evaluación por expertos, marco de trabajo SMART, Plan Estratégico para la Biodiversidad, post‐2020, Objetivos de Biodiversidad de Aichi

## Abstract

To inform governmental discussions on the nature of a revised Strategic Plan for Biodiversity of the Convention on Biological Diversity (CBD), we reviewed the relevant literature and assessed the framing of the 20 Aichi Biodiversity Targets in the current strategic plan. We asked international experts from nongovernmental organizations, academia, government agencies, international organizations, research institutes, and the CBD to score the Aichi Targets and their constituent elements against a set of specific, measurable, ambitious, realistic, unambiguous, scalable, and comprehensive criteria (SMART based, excluding time bound because all targets are bound to 2015 or 2020). We then investigated the relationship between these expert scores and reported progress toward the target elements by using the findings from 2 global progress assessments (Global Biodiversity Outlook and the Intergovernmental Science‐Policy Platform on Biodiversity and Ecosystem Services). We analyzed the data with ordinal logistic regressions. We found significant positive relationships (*p* < 0.05) between progress and the extent to which the target elements were perceived to be measurable, realistic, unambiguous, and scalable. There was some evidence of a relationship between progress and specificity of the target elements, but no relationship between progress and ambition. We are the first to show associations between progress and the extent to which the Aichi Targets meet certain SMART criteria. As negotiations around the post‐2020 biodiversity framework proceed, decision makers should strive to ensure that new or revised targets are effectively structured and clearly worded to allow the translation of targets into actionable policies that can be successfully implemented nationally, regionally, and globally.

## Abstract 2

*Article impact statement*: A post‐2020 framework for biodiversity conservation must ensure targets are clearly structured and worded to allow translation into policy.

## Abstract (es)

Relación de las Características de los Objetivos Mundiales de Biodiversidad con el Progreso Reportado

**Resumen**

Para informar las discusiones gubernamentales sobre la naturaleza de una revisión del Plan Estratégico para la Biodiversidad del Convenio sobre la Diversidad Biológica (CBD, en inglés), revisamos la literatura relevante y evaluamos el marco de 20 Objetivos de Biodiversidad de Aichi en el plan estratégico actual. Le pedimos a expertos internacionales de organizaciones no gubernamentales, de la academia, de agencias gubernamentales, organizaciones internacionales, de institutos de investigación y de la CBD que puntuaran los Objetivos de Aichi y sus elementos constituyentes frente a un conjunto de criterios específicos, medibles, ambiciosos, realistas (basados en SMART [las iniciales en inglés] y excluyendo aquellos limitados por el tiempo, pues todos los objetivos están limitados al 2015 o al 2020), inequívocos, expansibles y completos (excluyendo aquellos limitados por el tiempo). Después investigamos la relación entre los puntajes de estos expertos y el progreso reportado hacia los elementos objetivo usando los resultados de dos valoraciones mundiales del progreso (el Pronóstico Mundial de la Biodiversidad y la Plataforma Intergubernamental de Ciencia y Política sobre la Biodiversidad y los Servicios Ambientales). Analizamos los datos con regresiones logísticas ordinales. Encontramos relaciones positivas significativas (*p* < 0.05) entre el progreso y el alcance al que fueron percibidos como medibles, realistas, inequívocos y expansibles los elementos objetivo. Hubo algo de evidencia de la relación entre el progreso y la ambición. Somos los primeros en mostrar las asociaciones entre el progreso y la extensión hasta la que los Objetivos de Aichi cumplen con ciertos criterios SMART. Conforme proceden las negociaciones en torno al marco de trabajo de biodiversidad post‐2020, quienes toman las decisiones deberían esforzarse por asegurar que los objetivos nuevos o revisados estén estructurados efectivamente y redactados claramente para permitir la traducción de los objetivos hacia políticas factibles que puedan implementarse exitosamente a nivel nacional, regional y mundial.

## Introduction

The Strategic Plan for Biodiversity 2011–2020 of the Convention on Biological Diversity (CBD) is designed to provide an overarching framework on biodiversity conservation for the United Nations (UN) and other partners engaged in biodiversity management and policy development. The plan relates directly to the UN's Sustainable Development Goals, particularly, but not exclusively, to the 2 goals focused on the protection of marine and terrestrial life. The strategic plan includes 20 Aichi Biodiversity Targets (hereafter, Aichi Targets) organized into 5 strategic goals that aim to address the underlying causes of biodiversity loss by mainstreaming biodiversity across government and society (goal A); reduce the direct pressures on biodiversity and promote sustainable use (B); improve the status of biodiversity by safeguarding ecosystems, species, and genetic diversity (C); enhance the benefits to all from biodiversity and ecosystem services (D); and enhance implementation through participatory planning, knowledge management, and capacity building (E) (CBD 2010a). Parties to the CBD agreed to translate the Aichi Targets into national biodiversity strategies and action plans, and report against these at regular intervals. Progress to date has shown that, although a small number of targets are on track to be achieved (e.g., Target 16 on the Nagoya Protocol), there has been insufficient progress toward most of them or there has been movement away from the target (e.g., Target 14 on the restoration and safeguarding of ecosystems [Secretariat of the CBD 2014; Tittensor et al. 2014]). Progress is likely affected by many factors, including target tractability, alignment with existing governance structures and national priorities, financial and sociopolitical costs, and availability of resources. The framing and wording of the targets could also influence the ability of governments to interpret and translate them into policies and actions. For example, lack of a definition (until 2018) of “other effective area‐based conservation measures” under Target 11 has prevented governments from assessing their current coverage and implementing policies to increase these as a complement to formal protected area networks.

The strategic plan and associated targets will be reviewed in 2020. With discussions on a post‐2020 biodiversity framework underway following the 14th meeting of the Conference of the Parties in 2018, it is crucial to assess whether certain characteristics of the Aichi Targets are associated with achieving success as opposed to stasis or reported failure. Few researchers have reviewed the framing and wording of the targets. Butchart et al. (2016) assessed each of the target elements in terms of quantification, redundancy, ambiguity, and complexity. Wood (2011) carried out a “SMART” assessment of Target 11 that considered the degree to which it is specific, measurable, achievable, realistic, and time bound. The SMART framework is useful because it guides the development and assessment of objectives against criteria associated with clear, actionable targets. *SMART* is an acronym of 5 criteria considered important for the framing of effective, structured targets in project management (Doran 1981). No such assessment has been carried out across all targets, despite the clear importance of understanding the relationship between target characteristics and success. We conducted a SMART‐based assessment of the Aichi Targets and investigated associations between target characteristics and reported progress.

We searched systematically the peer‐reviewed literature relating to the Aichi Targets to inform our assessment and quantified variation in research effort among the targets. We then assessed the framing of the Aichi Targets through a structured survey that required expert assessors to score target elements against a set of SMART‐based criteria. We examined the relationship between the framing of the target elements and progress made toward the target elements, as reported in the 4th edition of Global Biodiversity Outlook (Secretariat of the CBD 2014) and the second‐order draft of the Intergovernmental Science‐Policy Platform on Biodiversity and Ecosystem Services (IPBES) *Global Assessment* (IPBES 2018). We hypothesized that progress toward targets is positively correlated with increasing specificity, measurability, realism, unambiguity, and scalability. We expected ambitious targets to be more difficult to achieve and therefore negatively associated with realism and progress. We devised recommendations on the characteristics of future biodiversity targets.

## Methods

### Literature Review

We identified scientific literature relating to the Aichi Targets by searching the titles, abstracts, and keywords of publications since 2010 in Web of Science and Scopus on 19 October 2017 with the search string “*biodivers* ^*^ AND *target* ^*^ AND (*Aichi* OR *CBD* OR “*Convention on Biological Diversity*”).” We retained all articles, conference papers, notes, reviews, series, and short surveys but excluded book chapters and publications that did not refer to the Aichi Biodiversity Targets. Due to time and resource constraints, our review was restricted to publications in English (8 publications [∼2%] not published in English). For each target, we counted the number of publications that mentioned the target number or a specific component of the target (e.g., “Target 11,” “the 17% Target,” or “the Aichi Target on Protected Areas”). We additionally reviewed the literature to identify criteria associated with the structure and wording of targets.

### Identifying SMART‐Based Criteria

We used a SMART‐based framework in our expert assessment. Different versions exist, but we followed the criteria recommended by the Subsidiary Body for Scientific, Technical, and Technological Advice (SBSTTA) of the CBD (CBD 2010b) and defined the components of SMART as specific, measurable, ambitious, realistic, and time bound. We identified 3 additional criteria that could be useful when assessing the framing of global biodiversity targets: comprehensive, unambiguous, and scalable (Bridgewater 2011; Collen et al. 2013; Butchart et al. 2016; Heywood 2016). Comprehensive is applicable at the target level, whereas all other criteria were assessed for individual elements within each target (most targets consist of multiple elements covering different aspects of the topic addressed [Supporting Information; Tittensor et al. 2014]). We did not investigate the relationship between the target‐level criterion comprehensive and progress because we could not identify a mechanism for such an association. Time bound was not assessed because each of the Aichi Targets is explicitly bound to 2015 (targets 10, 16, and 17) or 2020 (all other targets). Thus, although our criteria were based on the conventional SMART framework, we adapted the definition to remove one criterion (time bound) and include 3 additional criteria (comprehensive, unambiguous, and scalable). The criteria we used are hereafter referred to as SMART criteria.

Criteria definitions were presented throughout the expert assessment. *Specific* was defined as the “target element sets out clear and well‐defined objectives (e.g., quantified percentages, precisely defined terms, etc.).” *Measurable* was defined as “progress toward the target element can be assessed using data already available or feasible to mobilize by 2020 (e.g., quantitative indicators exist or are realistic to produce by 2020).” *Ambitious* was defined as the “target element is ambitious and aims sufficiently high to achieve the overall mission to halt the loss of biodiversity.” *Realistic* was defined as the “target element can feasibly be achieved considering the time frame, practicalities, plausible funding, etc.” *Comprehensive* was defined as the “target covers all important aspects of the issue that it seeks to address.” *Unambiguous* was defined as the “target element is easy to understand and interpret with a single, clear definition.” *Scalable* was defined as the “target element is applicable at global, regional, and national scales.”

### Expert Assessors

We adopted a purposive sampling approach (e.g., Teddlie & Yu 2007). This approach involves the sampling of special or unique cases for a specific purpose. In our case, we aimed to sample individuals with an in‐depth knowledge of the field rather than conservation in general. This was done to target the individuals from whom we could learn most. We approached 2 groups of experts with experience with the targets: individuals from nongovernmental organizations, academia, government agencies, international organizations, and research institutes who worked on the Aichi Targets and all primary and secondary CBD and SBSTTA National Focal Points. This approach ensured we invited individuals from all regions of the globe and increased the potential to gather a wide range of opinions while ensuring assessors had a detailed knowledge of the targets. Of 441 invitees, 49 participated in our assessment (see Supporting Information for a breakdown by UN Regional Group).

### Scoring the Targets

We sent candidate assessors a link to the survey, executed on http://Surveymonkey.com (Surveymonkey, San Mateo, California) and asked them to score the targets and their elements against the criteria on a scale of 0–10. There was an additional don't‐know option. A score of 0 meant the target or element did not fit the criterion at all and a score of 10 meant the target or element completely fit the criterion. The elements used in our assessment matched those in the draft IPBES Global Assessment (Supporting Information). We asked assessors to score at least 5 targets (including all constituent elements). To limit response bias toward more popular targets, we randomized the order of the targets for each participant and did not allow questions to be skipped. We ran the survey from 9 January 2018 until 1 February 2018. This relatively short timeframe was necessary to provide feedback to policy makers for discussion at the SBSTTA meeting in July 2018. The survey text was assessed and approved by the Royal Society for the Protection of Bird's Human Ethics Committee. Survey questions are provided in Supporting Information.

### Data Analyses

Targets and constituent elements were scored by between 20 and 34 assessors. For each target‐ or element‐criterion combination (e.g., Target 1, comprehensive; element 1.1, specific), we calculated the median of the expert scores excluding don't‐know answers. We examined the correlation between the median score per element and the percentage of don't‐know answers per element. We then assessed the overall SMART scores per strategic goal (A–E) by averaging across the scores per target per strategic goal for each element‐level criterion.

To investigate the relationship between the framing of elements and progress, we took categories of progress per target element from the Global Biodiversity Outlook 4 (GBO‐4) (Secretariat of the CBD 2014) and from analysis for the IPBES Global Assessment (IPBES 2018). In GBO‐4, progress toward each element was assessed on a scale from 1 to 5 (1 least to 5 most progress). The IPBES assessment contains an updated assessment of progress that builds on Tittensor et al. (2014). In the second‐order draft, progress toward each element is scored as poor, moderate, or good. Permission to use these data was granted by the IPBES Publication Review Committee. See Supporting Information for the definitions of progress categories from both assessments. Of the 54 elements included in our survey, progress was evaluated as unknown for 9 elements in the IPBES assessment and 3 in GBO‐4 due to a lack of suitable data. We omitted these elements from our analyses. There are small differences between the GBO‐4 and IPBES assessments in the breakdown of the Aichi Targets into elements. Because we matched the elements in our survey to those in the IPBES assessment, 4 elements were removed from our analyses when modeling with progress categories from the GBO‐4 assessment. This left 45 elements for the IPBES assessment and 47 elements for the GBO‐4 assessment.

For visualization purposes, we grouped the elements into progress categories and plotted the median score per element for each of our SMART criteria for the GBO‐4 and IPBES progress assessments separately. We calculated the means of those median scores per progress category and plotted these over the medians. We then fitted univariate ordinal logistic regression models in R 3.3.2 (R Core Team 2016) with the ordinal package (Christensen 2015) for each element‐level criterion. The progress category per element was regressed on the median score per element and weighted by the reciprocal of the standard deviation of the expert scores for that element‐criterion combination. We ran the analyses twice with the progress categories from the IPBES and GBO‐4 assessments separately. We repeated the analyses with progress categories from the GBO‐4 assessment after merging progress categories 4 (on track to achieve target) and 5 (on track to exceed target) due to the small number of elements in each. Additionally, we examined the degree to which the targets were viewed as comprehensive.

## Results

### Literature Review

**Figure 1:**
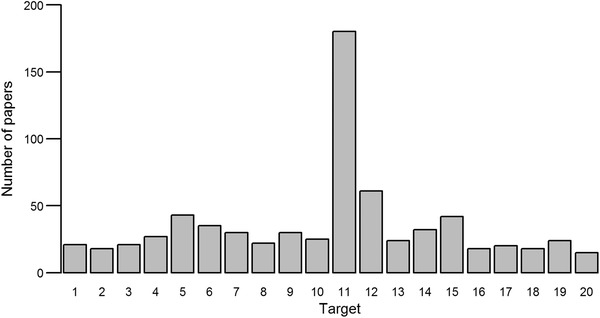
The number of papers identified from a literature search in Web of Science and Scopus that explicitly refer to each of the Aichi Targets. Some papers referred to more than one Target.

We identified 294 publications relevant to the Aichi Targets that we categorized into 6 research themes (Supporting Information). Research effort varied between targets. There was a large bias toward Target 11, on protected and conserved areas (Fig. 1 & Supporting Information), that was explicitly referred to in 61.2% of publications, nearly 3 times as many as the next‐most frequent target (Target 12, on preventing extinctions and conserving threatened species). The least mentioned was Target 20 (on mobilization of financial resources; explicitly referred to in just 5.1% of publications). Targets 2 (integration of biodiversity values into planning processes), 16 (the Nagoya Protocol), and 18 (traditional knowledge) were each explicitly referred to in just 6.1% of publications.

### Relating SMART Scores to Progress

**Figure 2:**
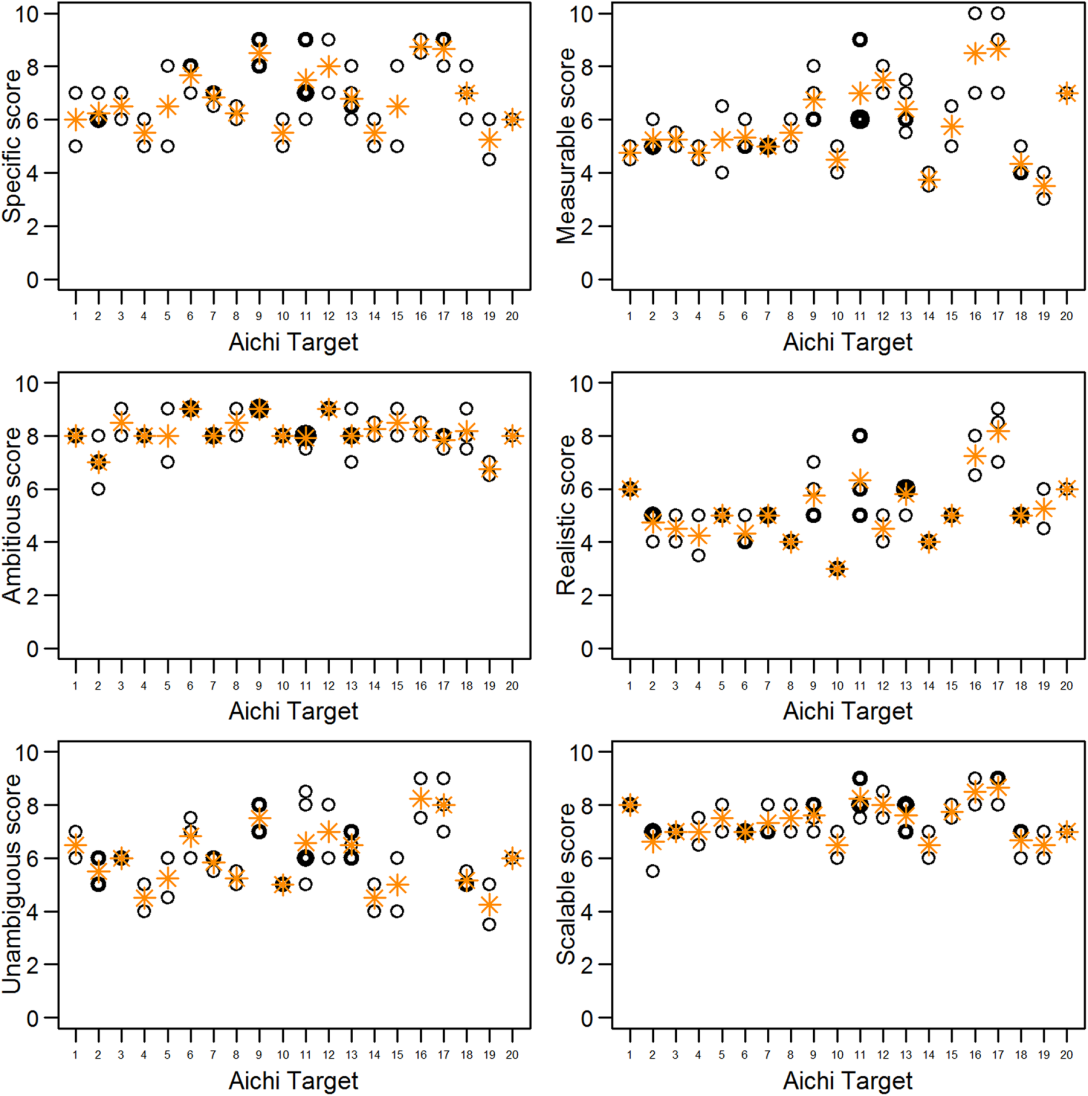
Median scores for each element of each Aichi Target (circles) and mean of the median scores for each target (asterisks) calculated from expert scores of the target elements against SMART‐based criteria (0, target element does not fit criterion at all; 10, target element completely fits criterion). Circle line width is a function of the number of overlapping median scores; thick lines indicate relatively more overlap between scores.

The percentage of don't‐know responses per target varied from 0% to 13.6%, with the highest percentages for Targets 16 and 18 and the lowest for Target 4 (Supporting Information). There was no relationship between the median score per element and the percentage of don't‐know responses for any criteria. All targets had moderate to high median scores for Comprehensive, ranging from 7 to 9.5 (Supporting Information). The element‐level scores for some SMART criteria were more variable than others. The median element‐level scores for ambitious and scalable were relatively consistent, whereas those for specific, measurable, realistic, and unambiguous were more variable (Fig. 2 & Supporting Information). There were significant positive correlations between many criteria (Supporting Information). The only negative relationship was between Ambitious and Realistic, but this was nonsignificant. Goal C had the highest overall SMART score per strategic goal, while goals A and B had the lowest (Supporting Information). Goal E included both the highest and lowest SMART scoring targets (17 and 19, respectively) (Supporting Information).

**Figure 3:**
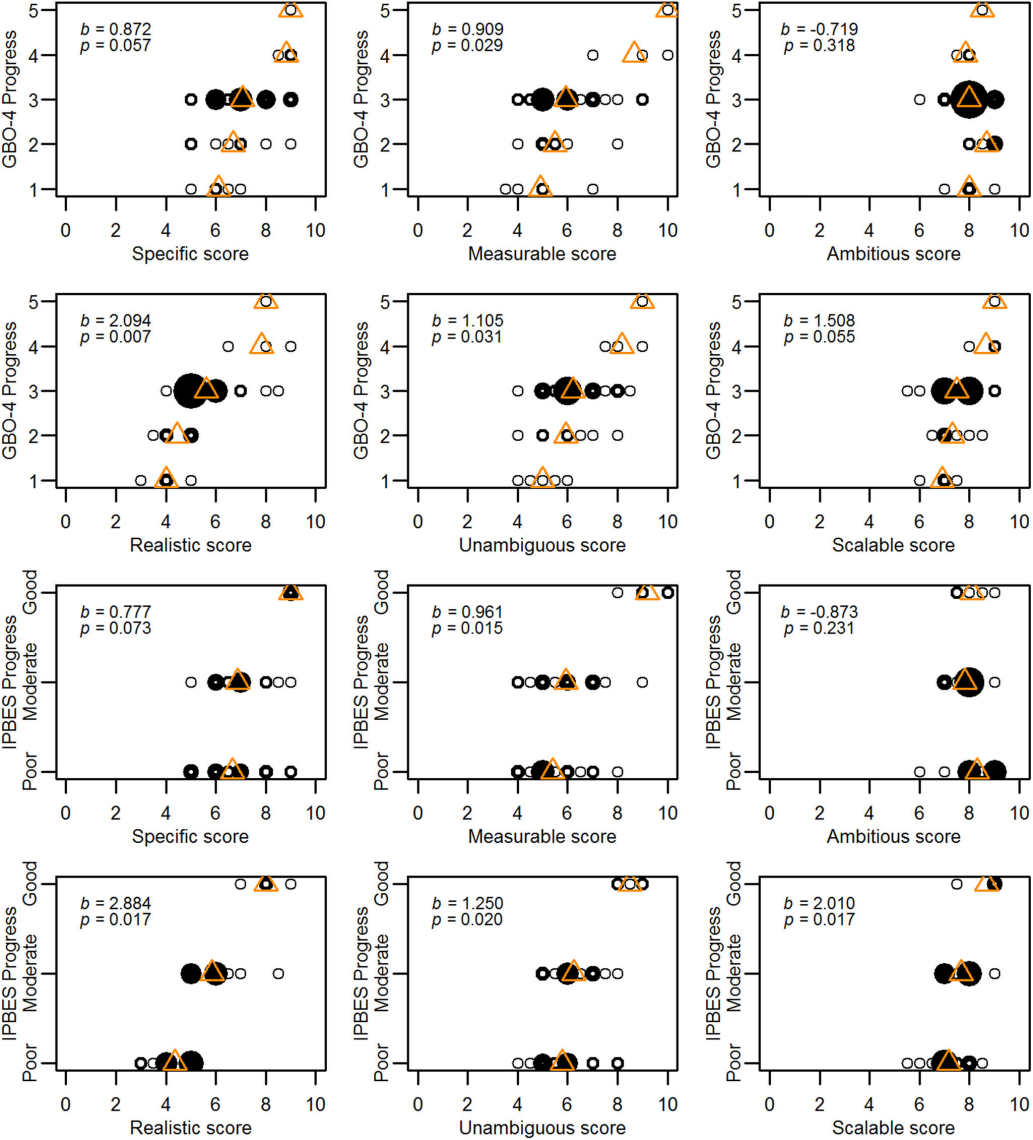
Expert scores of the Aichi Target elements against SMART‐based criteria (*x*‐axis) (0, target element does not fit criterion at all; 10, target element completely fits criterion) and progress toward the target elements from GBO‐4 and IPBES assessments (*y*‐axis) (GBO‐4, Global Biodiversity Outlook 4; IPBES, Intergovernmental Science‐Policy Platform on Biodiversity and Ecosystem Services Global Assessment) (circles, median expert score per element within each progress category; thick circle outline, relatively more overlap between median scores; triangles, mean of median scores per progress category; *b*, coefficients; *p*, *p*‐values from ordinal logistic regressions of progress category per element regressed on the median score per element per SMART criterion). In GBO‐4, progress toward each element was categorized on a scale from 1 to 5 (1 least to 5 most progress). In IPBES progress was categorized as poor, moderate, or good.

We found evidence of relationships between progress and some SMART criteria. Visualization of the data indicated positive relationships between progress and the degree to which our assessors considered different elements specific, measurable, realistic, unambiguous, or scalable (Fig. 3). Univariate ordinal logistic regressions identified significant positive relationships between progress and how measurable, realistic, unambiguous (GBO‐4 and IPBES assessments), and scalable (IPBES assessment only) the target elements were perceived to be (Table 1). Models with specific or unambiguous as the explanatory variable violated the proportional odds assumption of ordinal logistic regression when modeling with progress categories from the IPBES assessment. We therefore reanalyzed all criteria in multinomial logistic regressions with the progress categories from the IPBES assessment and found significant positive relationships between progress and scores for specific, measurable, realistic, unambiguous, and scalable (Supporting Information). We found no significant relationship between progress and scores for ambitious.

**Table 1:** Results of ordinal logistic regressions of categories of progress toward Aichi Biodiversity Target elements regressed on median scores of SMART criteria (second column) per target element.^a^

Progress assessment^b^	Criterion	Coefficient	SE	*Z*	*p*‐Value	Likelihood ratio	Likelihood ratio *p*
GBO‐4	specific	0.872 (0.869)	0.459 (0.458)	1.900 (1.898)	0.0574 (0.0577)	4.245 (4.23)	0.0394 (0.0397)
GBO‐4	unambiguous	1.105 (1.081)	0.511 (0.505)	2.162 (2.142)	0.0306 (0.0322)	5.791 (5.667)	0.0161 (0.0173)
GBO‐4	measurable	0.909 (0.884)	0.416 (0.411)	2.182 (2.150)	0.0291 (0.0316)	6.071 (5.889)	0.0138 (0.0152)
GBO‐4	ambitious	−0.719 (−0.723)	0.720 (0.719)	−0.998 (−1.005)	0.318 (0.315)	1.035 (1.050)	0.309 (0.306)
GBO‐4	realistic	2.094 (2.223)	0.778 (0.843)	2.691 (2.636)	0.0071 (0.0084)	15.19 (15.607)	<0.001 (<0.001)
GBO‐4	scalable	1.508 (1.498)	0.787 (0.785)	1.916 (1.909)	0.0554 (0.0563)	4.434 (4.399)	0.0352 (0.0360)
IPBES	specific	0.777	0.434	1.791	0.0732	3.624	0.0570
IPBES	unambiguous	1.250	0.539	2.318	0.0204	6.939	0.0084
IPBES	measurable	0.961	0.397	2.422	0.0154	7.661	0.0056
IPBES	ambitious	−0.873	0.728	−1.198	0.231	1.584	0.208
IPBES	realistic	2.884	1.205	2.393	0.0167	20.544	<0.001
IPBES	scalable	2.010	0.841	2.389	0.0169	7.323	0.0068

a Values in parentheses show results when progress categories 4 and 5 of the GBO‐4 assessment are combined.

b Abbreviations: GBO‐4, Global Biodiversity Outlook 4; IPBES, Intergovernmental Science‐Policy Platform on Biodiversity and Ecosystem Services Global Assessment.

## Discussion

Others have reflected on the framing of the Aichi Targets (Wood 2011; Butchart et al. 2016), but we are the first to carry out a SMART‐based assessment of all 20 targets and to show evidence of associations between progress and the extent to which the Aichi Targets were perceived to meet certain criteria. We found significant positive relationships between progress and measurability, unambiguity, realism and scalability of the target elements, and some evidence of a positive relationship with specificity. There was little variation between target elements in the scores for ambition, and we found no significant relationship between ambition and progress or realism. Because all Aichi Targets are explicitly time bound, this criterion was not assessed, but is an important characteristic when setting targets and should continue to be incorporated into any new target framework. Our results should be taken as indicative because they were based on the responses of 49 survey participants, most from the UN Regional Group's Western European and Others Group (Supporting Information). Although not feasible in this study, a longer time frame may have allowed a larger sample size with a more balanced geographical distribution and diverse set of opinions.

The mechanisms underpinning the relationship between target framing and progress were not explored here, but warrant further investigation. It is plausible that governments find it easier to identify and implement actions and policies if a target element is more specific and measurable and is unambiguously worded. Elements that are vague and open to interpretation may be more difficult to translate into specific actions or policies. Given that nearly all actions to address targets are applied nationally or locally, the association between progress and scalability of target elements is unsurprising. Similarly, the strong association between progress and target element realism is unsurprising: commitments that are unrealistic are less likely to be achieved.

Many factors will influence progress toward biodiversity targets. The midterm assessment by Tittensor et al. (2014) reported greater progress toward targets that covered responses to biodiversity loss than those targeting pressures on, states of, or benefits from biodiversity. This may be due to responses being easier to implement and measure than states and pressures and time lags between response implementation and consequent positive effects on biodiversity or the implemented responses being insufficient or inappropriate (Tittensor et al. 2014). The response targets (goal C) generally scored higher in our assessment than other targets, whereas targets focused on mainstreaming (goal A) and reducing pressures (goal B) scored lower. This indicates that targets related to the state of biodiversity and conservation responses to biodiversity loss were considered to more closely align with the SMART criteria used in our assessment than those targets relating to raising awareness, sustainable use, and pressure reduction, which strengthens the argument that responses are easier to action and measure. We found a large bias in research effort toward Target 11 (with a focus on protected areas in particular, a conservation response), which, in terms of terrestrial areal coverage, is on track to be achieved by 2020 (Secretariat of the CBD 2014). The area‐based elements of Target 11 had high SMART scores, whereas 4 qualitative elements (which call for “areas of particular importance for biodiversity and ecosystem services” to be conserved through “effectively and equitable managed, ecologically representative, and well‐connected systems of protected areas”) were perceived to be substantially less SMART (Supporting Information) and are unlikely to be achieved by 2020 (Secretariat of the CBD 2014). The mismatch in progress toward the more SMART quantitative elements and less SMART qualitative elements of Target 11 suggests the effectiveness of protected areas for conserving global biodiversity is currently limited by inappropriate placement and ineffective management. Protected areas often fail to capture ranges of threatened species (Beresford et al. 2011; Venter et al. 2014; Butchart et al. 2015) or are located in remote, mountainous areas that are under little pressure (Joppa & Pfaff 2011) and fail to halt the loss of natural land cover within their boundaries (Geldmann et al. 2013). Topography (elevation and slope) can be as effective at minimizing land cover change as designation of protected areas (Beresford et al. 2017).

The Aichi Targets have been described as more holistic and detailed, SMARTer, and better at addressing drivers of biodiversity loss than the CBD 2010 Biodiversity Target and other international targets (Harrop & Pritchard 2011; Collen et al. 2013; Mace et al. 2013; Harrop 2014; Rees et al. 2018). However, they have also been criticized for ambiguities, unquantified objectives, a lack of baselines and indicators, and insufficient objectives (Jørgensen 2013; Mace et al. 2013; Harris et al. 2014; Kadoya et al. 2014; Polak et al. 2015; Butchart et al. 2016; Heywood 2016; O'Leary et al. 2016; Beresford et al. 2017). Several studies suggest improvements that could be made when framing future biodiversity targets. These include ensuring that targets are SMART (Wood 2011; this paper) or meet other criteria, such as comprehensive, understandable, and enabling (Bridgewater 2011). Future frameworks could also consider employing a smaller number of more focused headline targets that are specific, quantified, simple, succinct, and unambiguous and that are accompanied by more specific subsidiary targets (Butchart et al. 2016). However, adhering to a SMART or similar framework during target setting, particularly around contentious issues, may be hindered unless sufficient focus is paid to the process of consensus building between stakeholders with diverse and often conflicting views (Maxwell et al. 2015). Targets should also reflect levels of conservation actions that are sufficient to deliver the protection and restoration of biodiversity, based on the best available scientific evidence (Di Marco et al. 2016). For example, to aim sufficiently high to halt the loss of biodiversity, there have been calls to increase the values of some quantitative elements, such as those of Target 11 (Locke 2013; O'Leary et al. 2016; Dinerstein et al. 2017; Krueck et al. 2017).

We recommend that any new or revised targets established under a post‐2020 global biodiversity framework should be clearly and unambiguously worded so that the intent and necessary action or actions are apparent; well defined with explicit deliverables and include quantifiable elements where appropriate so that progress toward the target can be measured; scalable so that actions can be implemented at global, national, and regional scales; and realistic so as to encourage action without being perceived as unachievable. See Supporting Information for an example with Target 7 (purely a demonstration of the application of a SMART framework). Although we cannot draw direct conclusions regarding ambition from our analyses, others have argued that targets need to aim sufficiently high to have positive outcomes for biodiversity and inspire action, but not so high that they become unrealistic to the point of reducing accountability and discouraging progress (Maxwell et al. 2015; Hagerman & Pelai 2016; Mace et al. 2018). We recognize that the target‐setting process is influenced by political practicalities and conflict resolution between diverse stakeholders (Larsen et al. 2015; Maxwell et al. 2015; Rodriguez‐Rodriguez et al. 2016). Nonetheless, Di Marco et al. (2016) called for scientists to be more engaged during target setting, specifically by providing policy makers with “direct evidence of how alternative formulations of targets […] can lead to improved biodiversity outcomes.” As negotiations around the post‐2020 global biodiversity framework proceed, we hope our analysis encourages decision makers to adhere to a SMART‐based framework as closely as possible to provide a strong basis for targets that are consistently interpreted, effectively applied, and adequate for the conservation, restoration, and sustainable use of biodiversity and ecosystem services.

## Supporting information

The elements of the Aichi Biodiversity Targets used in the expert assessment (Appendix S1), a breakdown of the number of assessors per UN Regional Group (Appendix S2), progress categories from GBO‐4 and IPBES Global Assessments (Appendix S3), research themes identified from a review of publications relating to the Aichi Targets (Appendix S4), median scores for comprehensive per target (Appendix S5), median scores per element per criterion (Appendix S6), correlation between scores for different criteria (Appendix S7), mean scores per target (Appendix S8), results of multinomial logistic regressions (Appendix S9), an example application of the SMART framework to Aichi Target 7 (Appendix S10), survey text used in the SMART assessment (Appendix S11), percentage of scores given as don't‐know per target (Appendix S12), and SMART scores per strategic goal (Appendix S13) are available online. The authors are solely responsible for the content and functionality of these materials. Queries (other than absence of the material) should be directed to the corresponding author.
