# Supplementary material for: Relating characteristics of global biodiversity targets to reported progress
Source: Conserv Biol. 2019 Jun 5;33(6):1360–9. doi: 10.1111/cobi.13322 (PMC6899758; doi:10.1111/cobi.13322)
Supplement: Supplementary file 1 — The elements of the Aichi Biodiversity Targets used in the expert assessment (Appendix S1), a breakdown of the number of assessors per UN Regional Group (Appendix S2), progress categories from GBO‐4 and IPBES Global Assessments (Appendix S3), research themes identified from a review of publications relating to the Aichi Targets (Appendix S4), median scores for comprehensive per target (Appendix S5), median scores per element per criterion (Appendix S6), correlation between scores for different criteria (Appendix S7), mean scores per target (Appendix S8), results of multinomial logistic regressions (Appendix S9), an example application of the SMART framework to Aichi Target 7 (Appendix S10), survey text used in the SMART assessment (Appendix S11), percentage of scores given as don't‐know per target (Appendix S12), and SMART scores per strategic goal (Appendix S13) are available online. The authors are solely responsible for the content and functionality of these materials. Queries (other than absence of the material) should be directed to the corresponding author. [file COBI-33-1360-s001.pdf]

Relating characteristics of global biodiversity targets to reported progress

Supporting Information

Appendix S1

The elements of the Aichi Biodiversity Targets used in the expert assessment, following the framework used in the IPBES *Global Assessment* (IPBES, 2018).

Target	Element no.	Element
Strategic Goal A: Address the underlying causes of biodiversity loss by mainstreaming biodiversity across government and society		
1	1.1	People are aware of the values of biodiversity
1	1.2	People are aware of […] the steps they can take to conserve and use it sustainably.
2	2.1	Biodiversity values have been integrated into national and local development and poverty reduction strategies
2	2.2	Biodiversity values have been […] integrated into national and local planning processes
2	2.3	Biodiversity values have been […] being incorporated into national accounting, as appropriate
2	2.4	Biodiversity values have been […] integrated into national […] reporting systems
3	3.1	Incentives, including subsidies, harmful to biodiversity are eliminated, phased out or reformed in order to minimize or avoid negative impacts
3	3.2	Positive incentives for the conservation and sustainable use of biodiversity are developed and applied, consistent and in harmony with the Convention and other relevant international obligations, taking into account national socio economic conditions.
4	4.1	Governments, business and stakeholders at all levels have taken steps to achieve or have implemented plans for sustainable production and consumption
4	4.2	Governments, business and stakeholders at all levels […] have kept the impacts of use of natural resources well within safe ecological limits.

Strategic Goal B: Reduce the direct pressures on biodiversity and promote sustainable use		
5	5.1	The rate of loss of all natural habitats, including forests, is at least halved and where feasible brought close to zero
5	5.2	Degradation and fragmentation [of natural habitats] is significantly reduced
6	6.1	All fish and invertebrate stocks and aquatic plants are managed and harvested sustainably, legally and applying ecosystem based approaches, overfishing is avoided [… and] the impacts of fisheries on stocks, species and ecosystems are within safe ecological limits
6	6.2	Recovery plans and measures are in place for all depleted species
6	6.3	Fisheries have no significant adverse impacts on threatened species and vulnerable ecosystems
7	7.1	Areas under agriculture […] are managed sustainably
7	7.2	Areas under aquaculture […] are managed sustainably
7	7.3	Areas under forestry […] are managed sustainably
8	8.1	Pollution […] has been brought to levels that are not detrimental to ecosystem function and biodiversity.
8	8.2	Pollution […] from excess nutrients should be brought to levels that are not detrimental to ecosystem function and biodiversity
9	9.1	Invasive alien species are identified and prioritized
9	9.2	[Invasive alien] pathways are identified and prioritized
9	9.3	Priority [invasive] species are controlled or eradicated
9	9.4	Measures are in place to manage pathways to prevent their introduction and establishment
10	10.1	The multiple anthropogenic pressures on coral reefs […] are minimized, so as to maintain their integrity and functioning
10	10.2	The multiple anthropogenic pressures on […] other vulnerable ecosystems impacted by climate change or ocean acidification are minimized, so as to maintain their integrity and functioning
Strategic Goal C: To improve the status of biodiversity by safeguarding ecosystems, species and genetic diversity		

11	11.1	At least 10 per cent of coastal and marine areas […] are conserved
11	11.2	At least 17 per cent of terrestrial and inland water areas […] are conserved
11	11.3	[…] Areas of particular importance for biodiversity and ecosystem services, are conserved
11	11.4	[Areas are conserved through] ecologically representative […] protected areas and other effective area-based conservation measures
11	11.5	[Areas are conserved through] effectively and equitably managed […] protected areas and other effective area-based conservation measures
11	11.6	[Areas are conserved through] well connected systems of protected areas and other effective area-based conservation measures and integrated into the wider landscapes and seascapes
12	12.1	The extinction of known threatened species has been prevented
12	12.2	The conservation status [of known threatened species, particularly of those most in decline] has been improved and sustained
13	13.1	The genetic diversity of cultivated plants […] is maintained
13	13.2	The genetic diversity of […] farmed and domesticated animals […] is maintained
13	13.3	The genetic diversity of […] wild relatives […]is maintained
13	13.4	The genetic diversity of […] socio-economically as well as culturally valuable species, is maintained
13	13.5	[…] Strategies have been developed and implemented for minimizing genetic erosion and safeguarding their genetic diversity
Strategic Goal D: Enhance the benefits to all from biodiversity and ecosystem services		
14	14.1	Ecosystems that provide essential services, including services related to water, and contributing to health, livelihoods and wellbeing, are restored and safeguarded
14	14.2	[…] Taking into account the needs of women, indigenous and local communities, and the poor and vulnerable
15	15.1	Ecosystem resilience and the contribution of biodiversity to carbon stocks has been enhanced, through conservation and restoration […] thereby contributing to climate change mitigation and adaptation and to combating desertification
15	15.2	[…] Including restoration of at least 15 per cent of degraded ecosystems […]

16	16.1	The Nagoya Protocol on Access to Genetic Resources and the Fair and EquiAppendix Sharing of Benefits Arising from their Utilization is in force [by 2015]
16	16.2	The Nagoya Protocol […] is operational [and] consistent with national legislation [by 2015]
Strategic Goal E: Enhance implementation through participatory planning, knowledge management and capacity building		
17	17.1	Each Party has developed[…] an effective, participatory and updated national biodiversity strategy and action plan (NBSAP)
17	17.2	Each Party has […] adopted as a policy instrument […] an effective, participatory and updated national biodiversity strategy and action plan (NBSAP)
17	17.3	Each Party has […] commenced implementing an effective, participatory and updated national biodiversity strategy and action plan (NBSAP)
18	18.1	The traditional knowledge, innovations and practices of indigenous and local communities relevant for the conservation and sustainable use of biodiversity, and their customary use of biological resources, are respected, subject to national legislation and relevant international obligations [...] at all relevant levels.
18	18.2	The traditional knowledge, innovations and practices of indigenous and local communities relevant for the conservation and sustainable use of biodiversity, and their customary use of biological resources, are [...] fully integrated and reflected in the implementation of the Convention [...] at all relevant levels.
18	18.3	The traditional knowledge, innovations and practices of indigenous and local communities relevant for the conservation and sustainable use of biodiversity, and their customary use of biological resources, [are respected, integrated, and reflected] with the full and effective participation of indigenous and local communities, at all relevant levels.
19	19.1	The science base and technologies relating to biodiversity, its values, functioning, status and trends, and the consequences of its loss, are improved, widely shared and transferred […]
19	19.2	The science base and technologies relating to biodiversity, its values, functioning, status and trends, and the consequences of its loss, are […] applied.

20	20.1	The mobilization of financial resources for effectively implementing the Strategic Plan for Biodiversity 2011-2020 from all sources, and in accordance with the consolidated and agreed process in the Strategy for Resource Mobilization, should increase substantially from the current levels [...]

Appendix S2

The number of assessors per United Nations Regional Group.

UN Regional Group	Number of assessors
African Group	4
Asia-Pacific	10
Eastern European Group	3
Latin American and Caribbean Group	3
Western European and Others Group	29

Appendix S3

Progress categories used in the assessment of the relationship between the framing of the elements and progress

Assessment	Progress category	Definition
GBO4	1	Moving away from target (things are getting worse rather than better)
GBO4	2	No significant overall progress (overall, we are neither moving towards the target nor moving away from it)
GBO4	3	Progress towards target but at an insufficient rate (unless we increase our efforts the target will not be met by its deadline)
GBO4	4	On track to achieve target (if we continue on our current trajectory we expect to achieve the target by 2020)
GBO4	5	On track to exceed target (we expect to achieve the target before its deadline)
IPBES	Poor	Little or no progress towards target element, or movement away from target. While there may be local/national or case-specific successes and positive trends for some aspects, the overall global trend shows little or negative progress.
IPBES	Moderate	Moderate progress towards target element. Overall global trend is positive, but insubstantial or insufficient, or there may be substantial positive trends for some aspects of the element, but little or no progress for others, or the trends are positive in some geographic regions but not in others.
IPBES	Good	Good progress towards target element. Substantial positive trends at a global scale relating to most aspects of the element.

Appendix S4

Research themes identified from a review of publications in Scopus and Web of Science relating to the Aichi Targets. Publications could fall into a single category, multiple categories or none. The vast majority of publications (90%) were captured by at least one of these categories. Total papers = 294.

Research theme	N publications
Relationships between the Aichi Targets (e.g. synergies and trade-offs)	21
How to monitor progress towards the targets (e.g. methods or indicators that enable progress to be assessed)	74
Assessments of progress (e.g. quantification of PA coverage)	70
How to make progress (papers that identify challenges to making progress and/or opportunities for progress to be made)	145
Funding the targets	11
Strengths and weaknesses of the targets	29

Appendix S5

Median scores for Comprehensive per Target, based on survey data collected in our expert SMART-based assessment of the Aichi Targets

Target	Comprehensive
1	8
2	7
3	8
4	7
5	7
6	8.5
7	8
8	9
9	9
10	7
11	9
12	8
13	8
14	8
15	7
16	9.5
17	9
18	8
19	7.5
20	8

Appendix S6

The median (Med) and standard deviation of the raw scores (SD) for all element-level criteria for each element, based on survey data collected in our SMART-based expert assessment of the Aichi Targets

Element	Specific		Ambitious		Measurable		Realistic		Unambiguous		Scalable	
	Med	SD	Med	SD	Med	SD	Med	SD	Med	SD	Med	SD
1.1	5	2.863	8	2.386	4.5	3.293	6	2.971	6	2.795	8	2.608
1.2	7	2.558	8	2.094	5	2.835	6	2.569	7	2.115	8	2.001
2.1	6	2.583	7	2.505	5	2.716	5	2.749	5	2.717	7	2.658
2.2	6	2.425	7	2.454	5	2.647	5	2.528	6	2.575	7	2.534
2.3	6	2.552	8	2.571	5	2.538	4	2.637	5	2.644	5.5	3.154
2.4	7	2.509	6	2.571	6	2.518	5	2.561	6	2.476	7	2.740
3.1	7	2.328	9	2.103	5.5	2.566	4	2.155	6	2.440	7	2.672
3.2	6	2.500	8	2.324	5	2.434	5	1.991	6	2.707	7	2.576
4.1	5	2.651	8	3.293	4.5	3.010	5	2.405	4	2.982	7.5	2.709
4.2	6	2.671	8	2.728	5	2.848	3.5	2.630	5	2.529	6.5	2.709
5.1	8	1.853	9	2.177	6.5	2.221	5	2.307	6	2.436	8	2.286
5.2	5	2.492	7	2.709	4	2.612	5	2.596	4.5	2.755	7	2.768
6.1	8	1.936	9	1.928	5	2.471	4	2.812	7	2.556	7	2.327
6.2	8	1.765	9	2.261	6	2.287	5	2.401	7.5	2.638	7	2.405
6.3	7	2.081	9	1.987	5	2.433	4	2.401	6	2.807	7	2.697
7.1	6.5	2.632	8	2.620	5	2.728	5	2.743	5.5	2.784	7	2.765
7.2	7	2.801	8	2.514	5	2.594	5	2.380	6	2.820	8	2.831
7.3	7	2.931	8	2.800	5	3.003	5	2.609	6	2.850	7	3.029
8.1	6	2.649	9	2.236	6	2.861	4	2.637	5	2.893	8	2.707
8.2	6.5	2.558	8	2.511	5	2.993	4	2.183	5.5	2.756	7	2.779
9.1	9	1.769	9	1.728	8	2.143	7	2.076	8	1.857	7.5	2.325
9.2	8	2.127	9	2.175	6	2.334	6	1.942	7	2.097	8	2.566
9.3	9	2.323	9	2.299	7	2.225	5	2.577	8	2.410	8	2.599
9.4	8	2.787	9	2.271	6	2.785	5	2.514	7	2.829	7	2.814
10.1	6	2.711	8	2.458	5	2.582	3	2.439	5	2.847	7	2.415
10.2	5	3.224	8	2.701	4	2.968	3	2.686	5	3.050	6	2.867

11.1	9	2.208	7.5	2.463	9	1.717	8	2.363	8.5	2.662	9	1.956
11.2	9	2.334	8	2.630	9	1.998	8	1.978	8	2.619	9	1.953
11.3	6	2.768	8	2.433	6	2.993	6	2.394	6	2.648	7.5	2.606
11.4	7	2.226	8	2.363	6	2.804	6	2.460	5	2.393	8	2.214
11.5	7	2.526	8	1.970	6	2.619	5	2.312	6	2.647	8	2.044
11.6	7	2.380	8	1.875	6	2.667	5	2.276	6	2.655	8	1.851
12.1	9	2.030	9	2.183	8	2.122	5	3.066	8	2.534	8.5	2.273
12.2	7	2.391	9	2.325	7	2.430	4	2.639	6	2.565	7.5	2.559
13.1	8	2.003	8	2.229	7	2.671	6	2.331	7	2.022	8	2.609
13.2	7	2.418	8	2.301	7.5	2.656	6	2.556	7	1.934	8	2.557
13.3	6.5	2.939	9	2.577	5.5	2.911	5	2.660	6.5	2.502	8	2.894
13.4	6	3.037	8	2.485	6	2.638	6	2.308	6	2.262	7	2.716
13.5	6.5	2.487	7	2.577	6	2.566	6	2.236	6	2.539	7	2.615
14.1	5	2.980	8.5	2.389	4	2.845	4	2.435	5	2.976	7	3.039
14.2	6	3.005	8	2.668	3.5	2.884	4	2.633	4	3.051	6	3.193
15.1	5	2.936	8	2.654	5	2.963	5	2.625	4	3.033	7.5	3.296
15.2	8	2.396	9	2.114	6.5	2.355	5	2.429	6	2.514	8	2.687
16.1	9	2.829	8.5	3.221	10	3.103	8	3.174	9	3.037	9	3.467
16.2	8.5	2.460	8	3.025	7	2.541	6.5	2.607	7.5	2.812	8	2.887
17.1	9	2.016	7.5	3.211	10	2.321	9	1.879	9	2.273	9	2.834
17.2	9	2.644	8	2.948	9	2.401	8.5	1.907	8	2.394	9	2.586
17.3	8	2.002	8	2.840	7	2.206	7	2.109	7	2.383	8	2.528
18.1	7	2.485	7.5	2.933	4	3.036	5	2.534	5.5	2.788	7	3.201
18.2	6	2.925	8	2.875	4	2.982	5	2.712	5	2.891	6	3.342
18.3	8	2.298	9	2.323	5	2.814	5	2.467	5	2.470	7	2.842
19.1	6	2.918	7	2.299	4	2.823	6	2.266	5	2.744	7	2.653
19.2	4.5	3.223	6.5	2.705	3	2.670	4.5	2.458	3.5	2.855	6	2.674
20.1	6	2.720	8	2.949	7	2.722	6	2.859	6	2.986	7	3.140

Appendix S7

Pearson's *r* correlation coefficient and two-tailed p-values showing the correlations between all element-level criteria

	Specific	Measurable	Ambitious	Realistic	Unambiguous	Scalable
Specific						
Measurable	*r* = 0.798, p <0.001					
Ambitious	*r* = 0.394, p = 0.003	*r* = 0.213, p = 0.122				
Realistic	*r* = 0.575, p <0.001	*r* = 0.749, p <0.001	*r* = -0.158, p = 0.255			
Unambiguous	*r* = 0.865, p <0.001	*r* = 0.855, p <0.001	*r* = 0.290, p = 0.034	*r* = 0.686, p <0.001		
Scalable	*r* = 0.654, p <0.001	*r* = 0.790, p <0.001	*r* = 0.206, p = 0.136	*r* = 0.714, p <0.001	*r* = 0.713, p <0.001	

Appendix S8

Mean scores per criterion per Target and mean scores across all criteria per Target

Goal	Target	Specific	Measurable	Ambitious	Realistic	Unambiguous	Scalable	Overall average
A	1	6.00	4.75	8.00	6.00	6.50	8.00	6.54
	2	6.25	5.25	7.00	4.75	5.50	6.63	5.90
	3	6.50	5.25	8.50	4.50	6.00	7.00	6.29
	4	5.50	4.75	8.00	4.25	4.50	7.00	5.67
B	5	6.50	5.25	8.00	5.00	5.25	7.50	6.25
	6	7.67	5.33	9.00	4.33	6.83	7.00	6.69
	7	6.83	5.00	8.00	5.00	5.83	7.33	6.33
	8	6.25	5.50	8.50	4.00	5.25	7.50	6.17
	9	8.50	6.75	9.00	5.75	7.50	7.63	7.52
	10	5.50	4.50	8.00	3.00	5.00	6.50	5.42
C	11	7.50	7.00	7.92	6.33	6.58	8.25	7.26
	12	8.00	7.50	9.00	4.50	7.00	8.00	7.33
	13	6.80	6.40	8.00	5.80	6.50	7.60	6.85
D	14	5.50	3.75	8.25	4.00	4.50	6.50	5.42
	15	6.50	5.75	8.50	5.00	5.00	7.75	6.42
	16	8.75	8.50	8.25	7.25	8.25	8.50	8.25
E	17	8.67	8.67	7.83	8.17	8.00	8.67	8.33
	18	7.00	4.33	8.17	5.00	5.17	6.67	6.06
	19	5.25	3.50	6.75	5.25	4.25	6.50	5.25
	20	6.00	7.00	8.00	6.00	6.00	7.00	6.67

Appendix S9

Results of multinomial logistic regressions using the package ‘nnet’ in R (R Core Team, 2016) where IPBES progress categories per Target element were regressed on median scores per Target element, with the reciprocal of the standard deviation of the raw scores fitted as a weight. The coefficients show the change in log odds of an element being in the “moderate” or “good” progress categories compared to the baseline “poor” category with a one-unit increase in the median score of the respective criterion.

Criterion	Progress category	Coefficient	SE	Likelihood-ratio test statistic	Likelihood-ratio test p-value
Specific	Moderate	0.1203	0.4563	9.1648	0.0102
	Good	7.3364	11.9860		
Unambiguous	Moderate	0.5198	0.5730	10.611	0.0050
	Good	8.5175	15.7730		
Measurable	Moderate	0.3854	0.4628	10.695	0.0048
	Good	2.7880	1.7372		
Ambitious	Moderate	-1.3349	0.8997	2.8121	0.2451
	Good	-0.4747	1.2790		
Realistic	Moderate	6.8160	11.1967	21.836	<0.001
	Good	8.7415	11.2631		
Scalable	Moderate	1.2695	0.9456	8.006	0.01826
	Good	4.1555	2.0690		

Appendix S10

An example of how the SMART framework could be applied to biodiversity targets, for illustrative purposes only.

Target no.	Target as written in the Strategic Plan 2011-2020	SMART example	Reasoning
7	By 2020 areas under agriculture, aquaculture and forestry are managed sustainably, ensuring conservation of biodiversity	By 2020 areas under agriculture, aquaculture and forestry are managed sustainably, as demonstrated by stable or increasing population trends of representative sets of species characteristic of these habitats.	Adding the means by which sustainable management of these production systems is to be demonstrated makes this target more Specific, Measurable and Unambiguous.

Appendix S11

Survey text used in the SMART assessment of the Aichi Targets.

Assessing the adequacy and effectiveness of the Aichi Biodiversity Targets

Thank you for participating in our survey. We are investigating the adequacy and effectiveness of the Aichi Targets in order to identify their strengths and weaknesses and inform future target setting.

This survey contains 20 pages with a different Target presented on each page. The order of the pages is randomised for each participant. You are unable to skip pages until you have answered all questions on the current page.

You do not need to complete the entire survey, but please score at least 5 Targets.When you wish to stop scoring, click "Save and Next" to save and submit your answers on the current page and then click "Exit" or leave the webpage. If you wish, you can return to the survey at anytime before the closing date and continue scoring from your last save point by re-opening the link in the email (you must use the same device and web browser throughout scoring).

Please read the instructions given at the top of each page carefully.

The survey will close at 17:30 (GMT) on February 1st 2018.

Your full name

[ ]

Your email address

[ ]

Do you give us permission to acknowledge your input in resulting publications and other outputs? This does not affect your participation in the survey.

○ Yes, I am happy to be acknowledged for my input

○ No, I do not want to be acknowledged

Assessing the adequacy and effectiveness of the Aichi Biodiversity Targets

Target 1

Please score the Target/elements presented on this page from 0 to 10 for the criteria listed below. One criterion applies to the overall Target ("Comprehensive"); the others apply to individual elements of the Target.

You must provide a score for every row before being able to move onto the next page. Your answers are saved when you click "Save and Next". To leave the page without saving, click the "Exit" button in the top right-hand corner of the page.

A score of 0 means the Target/element does not fit the criterion at all.
A score of 10 means the Target/element completely fits the criterion.

<u>Criteria</u>

- **Comprehensive: the Target covers all important aspects of the issue that it seeks to address**
- **Specific: the Target element sets out clear and well-defined objectives (e.g. quantified percentages, precisely defined terms etc)**
- **Ambitious: the Target element is ambitious and aims sufficiently high to achieve the overall mission to halt the loss of biodiversity**
- **Measurable: progress towards the Target element can be assessed using data already available or feasible to mobilise by 2020 (e.g. quantitative indicators exist or are realistic to produce by 2020)**
- **Realistic: the Target element can feasibly be achieved considering time-frame, practicalities, plausible funding etc**
- **Unambiguous: the Target element is easy to understand and interpret with a single, clear definition**
- **Scalable: the Target element is applicable at global, regional and national scales**

Scroll down or click "OK" to begin scoring this page

Target 1:
"By 2020, at the latest, people are aware of the values of biodiversity and the steps they can take to conserve and use it sustainably."

	0	1	2	3	4	5	6	7	8	9	10	Don't know
Comprehensive	○	○	○	○	○	○	○	○	○	○	○	○

Element 1.1:
"By 2020, [. . .] people are aware of the values of biodiversity"

	0	1	2	3	4	5	6	7	8	9	10	Don't know
Specific	○	○	○	○	○	○	○	○	○	○	○	○
Ambitious	○	○	○	○	○	○	○	○	○	○	○	○
Measurable	○	○	○	○	○	○	○	○	○	○	○	○
Realistic	○	○	○	○	○	○	○	○	○	○	○	○
Unambiguous	○	○	○	○	○	○	○	○	○	○	○	○
Scalable	○	○	○	○	○	○	○	○	○	○	○	○

Element 1.2:
"By 2020, [. . .] people are aware of [. . .] the steps they can take to conserve and use [biodiversity] sustainably"

	0	1	2	3	4	5	6	7	8	9	10	Don't know
Specific	○	○	○	○	○	○	○	○	○	○	○	○
Ambitious	○	○	○	○	○	○	○	○	○	○	○	○
Measurable	○	○	○	○	○	○	○	○	○	○	○	○
Realistic	○	○	○	○	○	○	○	○	○	○	○	○
Unambiguous	○	○	○	○	○	○	○	○	○	○	○	○
Scalable	○	○	○	○	○	○	○	○	○	○	○	○

Comments (optional)

Assessing the adequacy and effectiveness of the Aichi Biodiversity Targets

Target 2

Please score the Target/elements presented on this page from 0 to 10 for the criteria listed below. One criterion applies to the overall Target ("Comprehensive"); the others apply to individual elements of the Target.

You must provide a score for every row before being able to move onto the next page. Your answers are saved when you click "Save and Next". To leave the page without saving, click the "Exit" button in the top right-hand corner of the page.

A score of 0 means the Target/element does not fit the criterion at all.
A score of 10 means the Target/element completely fits the criterion.

Criteria

- **Comprehensive: the Target covers all important aspects of the issue that it seeks to address**
- **Specific: the Target element sets out clear and well-defined objectives (e.g. quantified percentages, precisely defined terms etc)**
- **Ambitious: the Target element is ambitious and aims sufficiently high to achieve the overall mission to halt the loss of biodiversity**
- **Measurable: progress towards the Target element can be assessed using data already available or feasible to mobilise by 2020 (e.g. quantitative indicators exist or are realistic to produce by 2020)**

- **Realistic: the Target element can feasibly be achieved considering time-frame, practicalities, plausible funding etc**
- **Unambiguous: the Target element is easy to understand and interpret with a single, clear definition**
- **Scalable: the Target element is applicable at global, regional and national scales**

Scroll down or click "OK" to begin scoring this page

Target 2:
"By 2020, at the latest, biodiversity values have been integrated into national and local development and poverty reduction strategies and planning processes and are being incorporated into national accounting, as appropriate, and reporting systems."

	0	1	2	3	4	5	6	7	8	9	10	Don't know
Comprehensive	○	○	○	○	○	○	○	○	○	○	○	○

Element 2.1:
"By 2020, [. . .] biodiversity values have been integrated into national and local **development and poverty reduction strategies**"

	0	1	2	3	4	5	6	7	8	9	10	Don't know
Specific	○	○	○	○	○	○	○	○	○	○	○	○
Ambitious	○	○	○	○	○	○	○	○	○	○	○	○
Measurable	○	○	○	○	○	○	○	○	○	○	○	○
Realistic	○	○	○	○	○	○	○	○	○	○	○	○
Unambiguous	○	○	○	○	○	○	○	○	○	○	○	○
Scalable	○	○	○	○	○	○	○	○	○	○	○	○

Element 2.2:
"By 2020, [. . .] biodiversity values have been integrated into national and local [. . .] **planning processes**"

	0	1	2	3	4	5	6	7	8	9	10	Don't know
Specific	○	○	○	○	○	○	○	○	○	○	○	○
Ambitious	○	○	○	○	○	○	○	○	○	○	○	○
Measurable	○	○	○	○	○	○	○	○	○	○	○	○
Realistic	○	○	○	○	○	○	○	○	○	○	○	○
Unambiguous	○	○	○	○	○	○	○	○	○	○	○	○
Scalable	○	○	○	○	○	○	○	○	○	○	○	○

Element 2.3:
"By 2020, [. . .] biodiversity values [. . .] are being incorporated into **national accounting**, as appropriate"

	0	1	2	3	4	5	6	7	8	9	10	Don't know
Specific												
Ambitious												
Measurable												
Realistic												
Unambiguous												
Scalable												

Element 2.4:
"By 2020, [. . .] biodiversity values [. . .] are being incorporated into **national** [. . .] **reporting systems**"

	0	1	2	3	4	5	6	7	8	9	10	Don't know
Specific												
Ambitious												
Measurable												
Realistic												
Unambiguous												
Scalable												

Comments (optional)

Target 3

Please score the Target/elements presented on this page from 0 to 10 for the criteria listed below. One criterion applies to the overall Target ("Comprehensive"); the others apply to individual elements of the Target.

You must provide a score for every row before being able to move onto the next page. Your answers are saved when you click "Save and Next". To leave the page without saving, click the "Exit" button in the top right-hand corner of the page.

A score of 0 means the Target/element does not fit the criterion at all.
A score of 10 means the Target/element completely fits the criterion.

Criteria

- **Comprehensive: the Target covers all important aspects of the issue that it seeks to address**
- **Specific: the Target element sets out clear and well-defined objectives (e.g. quantified percentages, precisely defined terms etc)**
- **Ambitious: the Target element is ambitious and aims sufficiently high to achieve the overall mission to halt the loss of biodiversity**
- **Measurable: progress towards the Target element can be assessed using data already available or feasible to mobilise by 2020 (e.g. quantitative indicators exist or are realistic to produce by 2020)**
- **Realistic: the Target element can feasibly be achieved considering time-frame, practicalities, plausible funding etc**
- **Unambiguous: the Target element is easy to understand and interpret with a single, clear definition**
- **Scalable: the Target element is applicable at global, regional and national scales**

Scroll down or click "OK" to begin scoring this page

Target 3:
"By 2020, at the latest, incentives, including subsidies, harmful to biodiversity are eliminated, phased out, or reformed in order to minimize or avoid negative impacts, and positive incentives for the conservation and sustainable use of biodiversity are developed and applied, consistent and in harmony with the convention and other relevant international obligations, taking into account national socioeconomic conditions."

	0	1	2	3	4	5	6	7	8	9	10	Don't know
Comprehensive	○	○	○	○	○	○	○	○	○	○	○	○

Element 3.1:
"By 2020, [. . .] incentives, including subsidies, harmful to biodiversity are eliminated, phased out, or reformed in order to minimize or avoid negative impacts"

	0	1	2	3	4	5	6	7	8	9	10	Don't know
Specific	○	○	○	○	○	○	○	○	○	○	○	○
Ambitious	○	○	○	○	○	○	○	○	○	○	○	○
Measurable	○	○	○	○	○	○	○	○	○	○	○	○
Realistic	○	○	○	○	○	○	○	○	○	○	○	○
Unambiguous	○	○	○	○	○	○	○	○	○	○	○	○
Scalable	○	○	○	○	○	○	○	○	○	○	○	○

Element 3.2:
"By 2020, [. . .] positive incentives for the conservation and sustainable use of biodiversity are developed and applied, consistent and in harmony with the convention and other relevant international obligations, taking into account national socioeconomic conditions."

	0	1	2	3	4	5	6	7	8	9	10	Don't know
Specific												
Ambitious												
Measurable												
Realistic												
Unambiguous												
Scalable												

Comments (optional)

Assessing the adequacy and effectiveness of the Aichi Biodiversity Targets

Target 4

Please score the Target/elements presented on this page from 0 to 10 for the criteria listed below. One criterion applies to the overall Target ("Comprehensive"); the others apply to individual elements of the Target.

You must provide a score for every row before being able to move onto the next page. Your answers are saved when you click "Save and Next". To leave the page without saving, click the "Exit" button in the top right-hand corner of the page.

A score of 0 means the Target/element does not fit the criterion at all.
A score of 10 means the Target/element completely fits the criterion.

<u>**Criteria**</u>

- **Comprehensive: the Target covers all important aspects of the issue that it seeks to address**
- **Specific: the Target element sets out clear and well-defined objectives (e.g. quantified percentages, precisely defined terms etc)**
- **Ambitious: the Target element is ambitious and aims sufficiently high to achieve the overall mission to halt the loss of biodiversity**
- **Measurable: progress towards the Target element can be assessed using data already available or feasible to mobilise by 2020 (e.g. quantitative indicators exist or are realistic to**

produce by 2020)

- **Realistic: the Target element can feasibly be achieved considering time-frame, practicalities, plausible funding etc**
- **Unambiguous: the Target element is easy to understand and interpret with a single, clear definition**
- **Scalable: the Target element is applicable at global, regional and national scales**

Scroll down or click "OK" to begin scoring this page

Target 4:

"By 2020, at the latest, Governments, business, and stakeholders at all levels have taken steps to achieve or have implemented plans for sustainable production and consumption and have kept the impacts of use of natural resources well within safe ecological limits."

	0	1	2	3	4	5	6	7	8	9	10	Don't know
Comprehensive	○	○	○	○	○	○	○	○	○	○	○	○

Element 4.1:

"By 2020, [. . .] Governments, business, and stakeholders at all levels have taken steps to achieve or have implemented plans for sustainable production and consumption"

	0	1	2	3	4	5	6	7	8	9	10	Don't know
Specific	○	○	○	○	○	○	○	○	○	○	○	○
Ambitious	○	○	○	○	○	○	○	○	○	○	○	○
Measurable	○	○	○	○	○	○	○	○	○	○	○	○
Realistic	○	○	○	○	○	○	○	○	○	○	○	○
Unambiguous	○	○	○	○	○	○	○	○	○	○	○	○
Scalable	○	○	○	○	○	○	○	○	○	○	○	○

Element 4.2:

"By 2020, [. . .] Governments, business, and stakeholders at all levels [. . .] have kept the impacts of use of natural resources well within safe ecological limits"

	0	1	2	3	4	5	6	7	8	9	10	Don't know
Specific	○	○	○	○	○	○	○	○	○	○	○	○
Ambitious	○	○	○	○	○	○	○	○	○	○	○	○
Measurable	○	○	○	○	○	○	○	○	○	○	○	○
Realistic	○	○	○	○	○	○	○	○	○	○	○	○
Unambiguous	○	○	○	○	○	○	○	○	○	○	○	○
Scalable	○	○	○	○	○	○	○	○	○	○	○	○

Comments (optional)

Assessing the adequacy and effectiveness of the Aichi Biodiversity Targets

Target 5

Please score the Target/elements presented on this page from 0 to 10 for the criteria listed below. One criterion applies to the overall Target ("Comprehensive"); the others apply to individual elements of the Target.

You must provide a score for every row before being able to move onto the next page. Your answers are saved when you click "Save and Next". To leave the page without saving, click the "Exit" button in the top right-hand corner of the page.

A score of 0 means the Target/element does not fit the criterion at all.
A score of 10 means the Target/element completely fits the criterion.

Criteria

- **Comprehensive: the Target covers all important aspects of the issue that it seeks to address**
- **Specific: the Target element sets out clear and well-defined objectives (e.g. quantified percentages, precisely defined terms etc)**
- **Ambitious: the Target element is ambitious and aims sufficiently high to achieve the overall mission to halt the loss of biodiversity**
- **Measurable: progress towards the Target element can be assessed using data already available or feasible to mobilise by 2020 (e.g. quantitative indicators exist or are realistic to produce by 2020)**
- **Realistic: the Target element can feasibly be achieved considering time-frame, practicalities, plausible funding etc**
- **Unambiguous: the Target element is easy to understand and interpret with a single, clear definition**
- **Scalable: the Target element is applicable at global, regional and national scales**

Scroll down or click "OK" to begin scoring this page

Target 5:
"By 2020, the rate of loss of all natural habitats, including forests, is at least halved and where feasible brought close to zero, and degradation and fragmentation is significantly reduced."

	0	1	2	3	4	5	6	7	8	9	10	Don't know
Comprehensive	○	○	○	○	○	○	○	○	○	○	○	○

Element 5.1:

"By 2020, the rate of loss of all natural habitats, including forests, is at least halved and where feasible brought close to zero"

	0	1	2	3	4	5	6	7	8	9	10	Don't know
Specific	○	○	○	○	○	○	○	○	○	○	○	○
Ambitious	○	○	○	○	○	○	○	○	○	○	○	○
Measurable	○	○	○	○	○	○	○	○	○	○	○	○
Realistic	○	○	○	○	○	○	○	○	○	○	○	○
Unambiguous	○	○	○	○	○	○	○	○	○	○	○	○
Scalable	○	○	○	○	○	○	○	○	○	○	○	○

Element 5.2:

"By 2020, [. . .] degradation and fragmentation [of natural habitats] is significantly reduced"

	0	1	2	3	4	5	6	7	8	9	10	Don't know
Specific	○	○	○	○	○	○	○	○	○	○	○	○
Ambitious	○	○	○	○	○	○	○	○	○	○	○	○
Measurable	○	○	○	○	○	○	○	○	○	○	○	○
Realistic	○	○	○	○	○	○	○	○	○	○	○	○
Unambiguous	○	○	○	○	○	○	○	○	○	○	○	○
Scalable	○	○	○	○	○	○	○	○	○	○	○	○

Comments (optional)

Assessing the adequacy and effectiveness of the Aichi Biodiversity Targets

Target 6

Please score the Target/elements presented on this page from 0 to 10 for the criteria listed below. One criterion applies to the overall Target ("Comprehensive"); the others apply to individual elements of the Target.

You must provide a score for every row before being able to move onto the next page. Your answers are saved when you click "Save and Next". To leave the page without saving, click the "Exit" button in the top right-hand corner of the page.

A score of 0 means the Target/element does not fit the criterion at all.
A score of 10 means the Target/element completely fits the criterion.

Criteria

- **Comprehensive: the Target covers all important aspects of the issue that it seeks to address**
- **Specific: the Target element sets out clear and well-defined objectives (e.g. quantified percentages, precisely defined terms etc)**
- **Ambitious: the Target element is ambitious and aims sufficiently high to achieve the overall mission to halt the loss of biodiversity**
- **Measurable: progress towards the Target element can be assessed using data already available or feasible to mobilise by 2020 (e.g. quantitative indicators exist or are realistic to produce by 2020)**
- **Realistic: the Target element can feasibly be achieved considering time-frame, practicalities, plausible funding etc**
- **Unambiguous: the Target element is easy to understand and interpret with a single, clear definition**
- **Scalable: the Target element is applicable at global, regional and national scales**

Scroll down or click "OK" to begin scoring this page

Target 6:
"By 2020, all fish and invertebrate stocks and aquatic plants are managed and harvested sustainably, legally, and applying ecosystem-based approaches, so that overfishing is avoided, recovery plans and measures are in place for all depleted species, fisheries have no significant adverse impacts on threatened species and vulnerable ecosystems, and the impacts of fisheries on stocks, species, and ecosystems are within safe ecological limits."

	0	1	2	3	4	5	6	7	8	9	10	Don't know
Comprehensive	○	○	○	○	○	○	○	○	○	○	○	○

Element 6.1:
"By 2020, all fish and invertebrate stocks and aquatic plants are managed and harvested sustainably, legally, and applying ecosystem-based approaches, so that overfishing is avoided [. . .] and the impacts of fisheries on stocks, species and ecosystems are within safe ecological limits"

	0	1	2	3	4	5	6	7	8	9	10	Don't know
Specific	○	○	○	○	○	○	○	○	○	○	○	○
Ambitious	○	○	○	○	○	○	○	○	○	○	○	○
Measurable	○	○	○	○	○	○	○	○	○	○	○	○
Realistic	○	○	○	○	○	○	○	○	○	○	○	○
Unambiguous	○	○	○	○	○	○	○	○	○	○	○	○
Scalable	○	○	○	○	○	○	○	○	○	○	○	○

Element 6.2:
"By 2020, [. . .] recovery plans and measures are in place for all depleted species"

	0	1	2	3	4	5	6	7	8	9	10	Don't know
Specific	◯	◯	◯	◯	◯	◯	◯	◯	◯	◯	◯	◯
Ambitious	◯	◯	◯	◯	◯	◯	◯	◯	◯	◯	◯	◯
Measurable	◯	◯	◯	◯	◯	◯	◯	◯	◯	◯	◯	◯
Realistic	◯	◯	◯	◯	◯	◯	◯	◯	◯	◯	◯	◯
Unambiguous	◯	◯	◯	◯	◯	◯	◯	◯	◯	◯	◯	◯
Scalable	◯	◯	◯	◯	◯	◯	◯	◯	◯	◯	◯	◯

Element 6.3:
"By 2020, [. . .] fisheries have no significant adverse impacts on threatened species and vulnerable ecosystems"

	0	1	2	3	4	5	6	7	8	9	10	Don't know
Specific	◯	◯	◯	◯	◯	◯	◯	◯	◯	◯	◯	◯
Ambitious	◯	◯	◯	◯	◯	◯	◯	◯	◯	◯	◯	◯
Measurable	◯	◯	◯	◯	◯	◯	◯	◯	◯	◯	◯	◯
Realistic	◯	◯	◯	◯	◯	◯	◯	◯	◯	◯	◯	◯
Unambiguous	◯	◯	◯	◯	◯	◯	◯	◯	◯	◯	◯	◯
Scalable	◯	◯	◯	◯	◯	◯	◯	◯	◯	◯	◯	◯

Comments (optional)

Assessing the adequacy and effectiveness of the Aichi Biodiversity Targets

Target 7

Please score the Target/elements presented on this page from 0 to 10 for the criteria listed below. One criterion applies to the overall Target ("Comprehensive"); the others apply to individual elements of the Target.

You must provide a score for every row before being able to move onto the next page. Your answers are saved when you click "Save and Next". To leave the page without saving, click the "Exit" button in the top right-hand corner of the page.

A score of 0 means the Target/element does not fit the criterion at all.
A score of 10 means the Target/element completely fits the criterion.

Criteria

- **Comprehensive: the Target covers all important aspects of the issue that it seeks to address**
- **Specific: the Target element sets out clear and well-defined objectives (e.g. quantified percentages, precisely defined terms etc)**
- **Ambitious: the Target element is ambitious and aims sufficiently high to achieve the overall mission to halt the loss of biodiversity**
- **Measurable: progress towards the Target element can be assessed using data already available or feasible to mobilise by 2020 (e.g. quantitative indicators exist or are realistic to produce by 2020)**
- **Realistic: the Target element can feasibly be achieved considering time-frame, practicalities, plausible funding etc**
- **Unambiguous: the Target element is easy to understand and interpret with a single, clear definition**
- **Scalable: the Target element is applicable at global, regional and national scales**

Scroll down or click "OK" to begin scoring this page

Target 7:
"By 2020, areas under agriculture, aquaculture, and forestry are managed sustainably, ensuring conservation of biodiversity."

	0	1	2	3	4	5	6	7	8	9	10	Don't know
Comprehensive	○	○	○	○	○	○	○	○	○	○	○	○

Element 7.1:
"By 2020, areas under **agriculture** [. . .] are managed sustainably"

	0	1	2	3	4	5	6	7	8	9	10	Don't know
Specific	○	○	○	○	○	○	○	○	○	○	○	○
Ambitious	○	○	○	○	○	○	○	○	○	○	○	○
Measurable	○	○	○	○	○	○	○	○	○	○	○	○
Realistic	○	○	○	○	○	○	○	○	○	○	○	○
Unambiguous	○	○	○	○	○	○	○	○	○	○	○	○
Scalable	○	○	○	○	○	○	○	○	○	○	○	○

Element 7.2:
"By 2020, areas under [. . .]**aquaculture** [. . .] are managed sustainably"

	0	1	2	3	4	5	6	7	8	9	10	Don't know
Specific	○	○	○	○	○	○	○	○	○	○	○	○
Ambitious	○	○	○	○	○	○	○	○	○	○	○	○
Measurable	○	○	○	○	○	○	○	○	○	○	○	○
Realistic	○	○	○	○	○	○	○	○	○	○	○	○
Unambiguous	○	○	○	○	○	○	○	○	○	○	○	○
Scalable	○	○	○	○	○	○	○	○	○	○	○	○

Element 7.3:
"By 2020, areas under [. . .]**forestry** are managed sustainably"

	0	1	2	3	4	5	6	7	8	9	10	Don't know
Specific	○	○	○	○	○	○	○	○	○	○	○	○
Ambitious	○	○	○	○	○	○	○	○	○	○	○	○
Measurable	○	○	○	○	○	○	○	○	○	○	○	○
Realistic	○	○	○	○	○	○	○	○	○	○	○	○
Unambiguous	○	○	○	○	○	○	○	○	○	○	○	○
Scalable	○	○	○	○	○	○	○	○	○	○	○	○

Comments (optional)

Assessing the adequacy and effectiveness of the Aichi Biodiversity Targets

Target 8

Please score the Target/elements presented on this page from 0 to 10 for the criteria listed below. One criterion applies to the overall Target ("Comprehensive"); the others apply to individual elements of the Target.

You must provide a score for every row before being able to move onto the next page. Your answers are saved when you click "Save and Next". To leave the page without saving, click the "Exit" button in the top right-hand corner of the page.

A score of 0 means the Target/element does not fit the criterion at all.

A score of 10 means the Target/element completely fits the criterion.

Criteria

- **Comprehensive: the Target covers all important aspects of the issue that it seeks to address**
- **Specific: the Target element sets out clear and well-defined objectives (e.g. quantified percentages, precisely defined terms etc)**
- **Ambitious: the Target element is ambitious and aims sufficiently high to achieve the overall mission to halt the loss of biodiversity**
- **Measurable: progress towards the Target element can be assessed using data already available or feasible to mobilise by 2020 (e.g. quantitative indicators exist or are realistic to produce by 2020)**
- **Realistic: the Target element can feasibly be achieved considering time-frame, practicalities, plausible funding etc**
- **Unambiguous: the Target element is easy to understand and interpret with a single, clear definition**
- **Scalable: the Target element is applicable at global, regional and national scales**

Scroll down or click "OK" to begin scoring this page

Target 8:
"By 2020, pollution, including from excess nutrients, has been brought to levels that are not detrimental to ecosystem function and biodiversity."

	0	1	2	3	4	5	6	7	8	9	10	Don't know
Comprehensive	○	○	○	○	○	○	○	○	○	○	○	○

Element 8.1:
"By 2020, pollution [. . .] has been brought to levels that are not detrimental to ecosystem function and biodiversity."

	0	1	2	3	4	5	6	7	8	9	10	Don't know
Specific	○	○	○	○	○	○	○	○	○	○	○	○
Ambitious	○	○	○	○	○	○	○	○	○	○	○	○
Measurable	○	○	○	○	○	○	○	○	○	○	○	○
Realistic	○	○	○	○	○	○	○	○	○	○	○	○
Unambiguous	○	○	○	○	○	○	○	○	○	○	○	○
Scalable	○	○	○	○	○	○	○	○	○	○	○	○

Element 8.2:
"By 2020, pollution [. . .]**from excess nutrients** has been brought to levels that are not detrimental to ecosystem function and biodiversity"

	0	1	2	3	4	5	6	7	8	9	10	Don't know
Specific	○	○	○	○	○	○	○	○	○	○	○	○
Ambitious	○	○	○	○	○	○	○	○	○	○	○	○
Measurable	○	○	○	○	○	○	○	○	○	○	○	○
Realistic	○	○	○	○	○	○	○	○	○	○	○	○
Unambiguous	○	○	○	○	○	○	○	○	○	○	○	○
Scalable	○	○	○	○	○	○	○	○	○	○	○	○

Comments (optional)

Assessing the adequacy and effectiveness of the Aichi Biodiversity Targets

Target 9

Please score the Target/elements presented on this page from 0 to 10 for the criteria listed below. One criterion applies to the overall Target ("Comprehensive"); the others apply to individual elements of the Target.

You must provide a score for every row before being able to move onto the next page. Your answers are saved when you click "Save and Next". To leave the page without saving, click the "Exit" button in the top right-hand corner of the page.

A score of 0 means the Target/element does not fit the criterion at all.
A score of 10 means the Target/element completely fits the criterion.

Criteria

- **Comprehensive: the Target covers all important aspects of the issue that it seeks to address**
- **Specific: the Target element sets out clear and well-defined objectives (e.g. quantified percentages, precisely defined terms etc)**
- **Ambitious: the Target element is ambitious and aims sufficiently high to achieve the overall mission to halt the loss of biodiversity**
- **Measurable: progress towards the Target element can be assessed using data already available or feasible to mobilise by 2020 (e.g. quantitative indicators exist or are realistic to produce by 2020)**

- **Realistic: the Target element can feasibly be achieved considering time-frame, practicalities, plausible funding etc**
- **Unambiguous: the Target element is easy to understand and interpret with a single, clear definition**
- **Scalable: the Target element is applicable at global, regional and national scales**

Scroll down or click "OK" to begin scoring this page

Target 9:
"By 2020, invasive alien species and pathways are identified and prioritized, priority species are controlled or eradicated, and measures are in place to manage pathways to prevent their introduction and establishment."

	0	1	2	3	4	5	6	7	8	9	10	Don't know
Comprehensive	○	○	○	○	○	○	○	○	○	○	○	○

Element 9.1:
"By 2020, **invasive alien species** [. . .] are identified and prioritized"

	0	1	2	3	4	5	6	7	8	9	10	Don't know
Specific	○	○	○	○	○	○	○	○	○	○	○	○
Ambitious	○	○	○	○	○	○	○	○	○	○	○	○
Measurable	○	○	○	○	○	○	○	○	○	○	○	○
Realistic	○	○	○	○	○	○	○	○	○	○	○	○
Unambiguous	○	○	○	○	○	○	○	○	○	○	○	○
Scalable	○	○	○	○	○	○	○	○	○	○	○	○

Element 9.2:
"By 2020, **invasive alien** [. . .]**pathways** are identified and prioritized"

	0	1	2	3	4	5	6	7	8	9	10	Don't know
Specific	○	○	○	○	○	○	○	○	○	○	○	○
Ambitious	○	○	○	○	○	○	○	○	○	○	○	○
Measurable	○	○	○	○	○	○	○	○	○	○	○	○
Realistic	○	○	○	○	○	○	○	○	○	○	○	○
Unambiguous	○	○	○	○	○	○	○	○	○	○	○	○
Scalable	○	○	○	○	○	○	○	○	○	○	○	○

Element 9.3:
"By 2020, [. . .] priority [invasive] species are controlled or eradicated"

	0	1	2	3	4	5	6	7	8	9	10	Don't know
Specific												
Ambitious												
Measurable												
Realistic												
Unambiguous												
Scalable												

Element 9.4:
"By 2020, [. . .] measures are in place to manage pathways to prevent their introduction and establishment"

	0	1	2	3	4	5	6	7	8	9	10	Don't know
Specific												
Ambitious												
Measurable												
Realistic												
Unambiguous												
Scalable												

Comments (optional)

Assessing the adequacy and effectiveness of the Aichi Biodiversity Targets

Target 10

Please score the Target/elements presented on this page from 0 to 10 for the criteria listed below. One criterion applies to the overall Target ("Comprehensive"); the others apply to individual elements of the Target.

You must provide a score for every row before being able to move onto the next page. Your answers are saved when you click "Save and Next". To leave the page without saving, click the "Exit" button in the top right-hand corner of the page.

A score of 0 means the Target/element does not fit the criterion at all.
A score of 10 means the Target/element completely fits the criterion.

Criteria

- **Comprehensive: the Target covers all important aspects of the issue that it seeks to address**
- **Specific: the Target element sets out clear and well-defined objectives (e.g. quantified percentages, precisely defined terms etc)**
- **Ambitious: the Target element is ambitious and aims sufficiently high to achieve the overall mission to halt the loss of biodiversity**
- **Measurable: progress towards the Target element can be assessed using data already available or feasible to mobilise by 2020 (e.g. quantitative indicators exist or are realistic to produce by 2020)**
- **Realistic: the Target element can feasibly be achieved considering time-frame, practicalities, plausible funding etc**
- **Unambiguous: the Target element is easy to understand and interpret with a single, clear definition**
- **Scalable: the Target element is applicable at global, regional and national scales**

Scroll down or click "OK" to begin scoring this page

Target 10:
"By 2015, the multiple anthropogenic pressures on coral reefs, and other vulnerable ecosystems impacted by climate change or ocean acidification are minimized, so as to maintain their integrity and functioning."

	0	1	2	3	4	5	6	7	8	9	10	Don't know
Comprehensive	○	○	○	○	○	○	○	○	○	○	○	○

Element 10.1:
"By 2015, the multiple anthropogenic pressures on**coral reefs** [. . .] are minimized, so as to maintain their integrity and functioning"

	0	1	2	3	4	5	6	7	8	9	10	Don't know
Specific	○	○	○	○	○	○	○	○	○	○	○	○
Ambitious	○	○	○	○	○	○	○	○	○	○	○	○
Measurable	○	○	○	○	○	○	○	○	○	○	○	○
Realistic	○	○	○	○	○	○	○	○	○	○	○	○
Unambiguous	○	○	○	○	○	○	○	○	○	○	○	○
Scalable	○	○	○	○	○	○	○	○	○	○	○	○

Element 10.2:
"By 2015, the multiple anthropogenic pressures on [. . .] **other vulnerable ecosystems** impacted by climate change or ocean acidification are minimized, so as to maintain their integrity and functioning"

	0	1	2	3	4	5	6	7	8	9	10	Don't know
Specific	◯	◯	◯	◯	◯	◯	◯	◯	◯	◯	◯	◯
Ambitious	◯	◯	◯	◯	◯	◯	◯	◯	◯	◯	◯	◯
Measurable	◯	◯	◯	◯	◯	◯	◯	◯	◯	◯	◯	◯
Realistic	◯	◯	◯	◯	◯	◯	◯	◯	◯	◯	◯	◯
Unambiguous	◯	◯	◯	◯	◯	◯	◯	◯	◯	◯	◯	◯
Scalable	◯	◯	◯	◯	◯	◯	◯	◯	◯	◯	◯	◯

Comments (optional)

Assessing the adequacy and effectiveness of the Aichi Biodiversity Targets

Target 11

Please score the Target/elements presented on this page from 0 to 10 for the criteria listed below. One criterion applies to the overall Target ("Comprehensive"); the others apply to individual elements of the Target.

You must provide a score for every row before being able to move onto the next page. Your answers are saved when you click "Save and Next". To leave the page without saving, click the "Exit" button in the top right-hand corner of the page.

A score of 0 means the Target/element does not fit the criterion at all.
A score of 10 means the Target/element completely fits the criterion.

Criteria

- **Comprehensive: the Target covers all important aspects of the issue that it seeks to address**
- **Specific: the Target element sets out clear and well-defined objectives (e.g. quantified percentages, precisely defined terms etc)**
- **Ambitious: the Target element is ambitious and aims sufficiently high to achieve the overall mission to halt the loss of biodiversity**
- **Measurable: progress towards the Target element can be assessed using data already available or feasible to mobilise by 2020 (e.g. quantitative indicators exist or are realistic to produce by 2020)**

- **Realistic: the Target element can feasibly be achieved considering time-frame, practicalities, plausible funding etc**
- **Unambiguous: the Target element is easy to understand and interpret with a single, clear definition**
- **Scalable: the Target element is applicable at global, regional and national scales**

Scroll down or click "OK" to begin scoring this page

Target 11:
"By 2020, at least 17 per cent of terrestrial and inland water, and 10 per cent of coastal and marine areas, especially areas of particular importance for biodiversity and ecosystem services, are conserved through effectively and equitably managed, ecologically representative and well connected systems of protected areas and other effective area-based conservation measures, and integrated into the wider landscapes and seascapes."

	0	1	2	3	4	5	6	7	8	9	10	Don't know
Comprehensive	○	○	○	○	○	○	○	○	○	○	○	○

Element 11.1:
"By 2020, at least [. . .] **10% of coastal and marine areas** [. . .] are conserved"

	0	1	2	3	4	5	6	7	8	9	10	Don't know
Specific	○	○	○	○	○	○	○	○	○	○	○	○
Ambitious	○	○	○	○	○	○	○	○	○	○	○	○
Measurable	○	○	○	○	○	○	○	○	○	○	○	○
Realistic	○	○	○	○	○	○	○	○	○	○	○	○
Unambiguous	○	○	○	○	○	○	○	○	○	○	○	○
Scalable	○	○	○	○	○	○	○	○	○	○	○	○

Element 11.2:
"By 2020, at least **17% of terrestrial and inland water areas** [. . .] are conserved"

	0	1	2	3	4	5	6	7	8	9	10	Don't know
Specific	○	○	○	○	○	○	○	○	○	○	○	○
Ambitious	○	○	○	○	○	○	○	○	○	○	○	○
Measurable	○	○	○	○	○	○	○	○	○	○	○	○
Realistic	○	○	○	○	○	○	○	○	○	○	○	○
Unambiguous	○	○	○	○	○	○	○	○	○	○	○	○
Scalable	○	○	○	○	○	○	○	○	○	○	○	○

Element 11.3

"By 2020, [. . .] areas of particular importance for biodiversity and ecosystem services, are conserved"

	0	1	2	3	4	5	6	7	8	9	10	Don't know
Specific	○	○	○	○	○	○	○	○	○	○	○	○
Ambitious	○	○	○	○	○	○	○	○	○	○	○	○
Measurable	○	○	○	○	○	○	○	○	○	○	○	○
Realistic	○	○	○	○	○	○	○	○	○	○	○	○
Unambiguous	○	○	○	○	○	○	○	○	○	○	○	○
Scalable	○	○	○	○	○	○	○	○	○	○	○	○

Element 11.4

"By 2020, [. . . areas are conserved through] **ecologically representative** [. . .] protected areas and other effective area-based conservation measures"

	0	1	2	3	4	5	6	7	8	9	10	Don't know
Specific	○	○	○	○	○	○	○	○	○	○	○	○
Ambitious	○	○	○	○	○	○	○	○	○	○	○	○
Measurable	○	○	○	○	○	○	○	○	○	○	○	○
Realistic	○	○	○	○	○	○	○	○	○	○	○	○
Unambiguous	○	○	○	○	○	○	○	○	○	○	○	○
Scalable	○	○	○	○	○	○	○	○	○	○	○	○

Element 11.5:

"By 2020, [. . . areas] are conserved through **effectively and equitably managed** [. . .] protected areas and other effective area-based conservation measures"

	0	1	2	3	4	5	6	7	8	9	10	Don't know
Specific	○	○	○	○	○	○	○	○	○	○	○	○
Ambitious	○	○	○	○	○	○	○	○	○	○	○	○
Measurable	○	○	○	○	○	○	○	○	○	○	○	○
Realistic	○	○	○	○	○	○	○	○	○	○	○	○
Unambiguous	○	○	○	○	○	○	○	○	○	○	○	○
Scalable	○	○	○	○	○	○	○	○	○	○	○	○

Element 11.6:
"By 2020, [. . . areas are conserved through] **well connected systems** of protected areas and other effective area-based conservation measures and integrated into the wider landscapes and seascapes"

	0	1	2	3	4	5	6	7	8	9	10	Don't know
Specific	◯	◯	◯	◯	◯	◯	◯	◯	◯	◯	◯	◯
Ambitious	◯	◯	◯	◯	◯	◯	◯	◯	◯	◯	◯	◯
Measurable	◯	◯	◯	◯	◯	◯	◯	◯	◯	◯	◯	◯
Realistic	◯	◯	◯	◯	◯	◯	◯	◯	◯	◯	◯	◯
Unambiguous	◯	◯	◯	◯	◯	◯	◯	◯	◯	◯	◯	◯
Scalable	◯	◯	◯	◯	◯	◯	◯	◯	◯	◯	◯	◯

Comments (optional)

Assessing the adequacy and effectiveness of the Aichi Biodiversity Targets

Target 12

Please score the Target/elements presented on this page from 0 to 10 for the criteria listed below. One criterion applies to the overall Target ("Comprehensive"); the others apply to individual elements of the Target.

You must provide a score for every row before being able to move onto the next page. Your answers are saved when you click "Save and Next". To leave the page without saving, click the "Exit" button in the top right-hand corner of the page.

A score of 0 means the Target/element does not fit the criterion at all.
A score of 10 means the Target/element completely fits the criterion.

Criteria

- **Comprehensive: the Target covers all important aspects of the issue that it seeks to address**
- **Specific: the Target element sets out clear and well-defined objectives (e.g. quantified percentages, precisely defined terms etc)**
- **Ambitious: the Target element is ambitious and aims sufficiently high to achieve the overall mission to halt the loss of biodiversity**
- **Measurable: progress towards the Target element can be assessed using data already available or feasible to mobilise by 2020 (e.g. quantitative indicators exist or are realistic to produce by 2020)**

- **Realistic: the Target element can feasibly be achieved considering time-frame, practicalities, plausible funding etc**
- **Unambiguous: the Target element is easy to understand and interpret with a single, clear definition**
- **Scalable: the Target element is applicable at global, regional and national scales**

Scroll down or click "OK" to begin scoring this page

Target 12:
"By 2020, the extinction of known threatened species has been prevented and their conservation status, particularly of those most in decline, has been improved and sustained."

	0	1	2	3	4	5	6	7	8	9	10	Don't know
Comprehensive	○	○	○	○	○	○	○	○	○	○	○	○

Element 12.1:
"By 2020, the extinction of known threatened species has been prevented"

	0	1	2	3	4	5	6	7	8	9	10	Don't know
Specific	○	○	○	○	○	○	○	○	○	○	○	○
Ambitious	○	○	○	○	○	○	○	○	○	○	○	○
Measurable	○	○	○	○	○	○	○	○	○	○	○	○
Realistic	○	○	○	○	○	○	○	○	○	○	○	○
Unambiguous	○	○	○	○	○	○	○	○	○	○	○	○
Scalable	○	○	○	○	○	○	○	○	○	○	○	○

Element 12.2:
"By 2020, [. . .] the conservation status [of known threatened species, particularly of those most in decline] has been improved and sustained"

	0	1	2	3	4	5	6	7	8	9	10	Don't know
Specific	○	○	○	○	○	○	○	○	○	○	○	○
Ambitious	○	○	○	○	○	○	○	○	○	○	○	○
Measurable	○	○	○	○	○	○	○	○	○	○	○	○
Realistic	○	○	○	○	○	○	○	○	○	○	○	○
Unambiguous	○	○	○	○	○	○	○	○	○	○	○	○
Scalable	○	○	○	○	○	○	○	○	○	○	○	○

Comments (optional)

Target 13

Please score the Target/elements presented on this page from 0 to 10 for the criteria listed below. One criterion applies to the overall Target ("Comprehensive"); the others apply to individual elements of the Target.

You must provide a score for every row before being able to move onto the next page. Your answers are saved when you click "Save and Next". To leave the page without saving, click the "Exit" button in the top right-hand corner of the page.

A score of 0 means the Target/element does not fit the criterion at all.
A score of 10 means the Target/element completely fits the criterion.

Criteria

- **Comprehensive: the Target covers all important aspects of the issue that it seeks to address**
- **Specific: the Target element sets out clear and well-defined objectives (e.g. quantified percentages, precisely defined terms etc)**
- **Ambitious: the Target element is ambitious and aims sufficiently high to achieve the overall mission to halt the loss of biodiversity**
- **Measurable: progress towards the Target element can be assessed using data already available or feasible to mobilise by 2020 (e.g. quantitative indicators exist or are realistic to produce by 2020)**
- **Realistic: the Target element can feasibly be achieved considering time-frame, practicalities, plausible funding etc**
- **Unambiguous: the Target element is easy to understand and interpret with a single, clear definition**
- **Scalable: the Target element is applicable at global, regional and national scales**

Scroll down or click "OK" to begin scoring this page

Target 13:
"By 2020, the genetic diversity of cultivated plants and farmed and domesticated animals and of wild relatives, including other socioeconomically as well as culturally valuable species, is maintained, and strategies have been developed and implemented for minimizing genetic erosion and safeguarding their genetic diversity."

	0	1	2	3	4	5	6	7	8	9	10	Don't know
Comprehensive	○	○	○	○	○	○	○	○	○	○	○	○

Element 13.1:

"By 2020, the genetic diversity of **cultivated plants** [. . .] is maintained"

	0	1	2	3	4	5	6	7	8	9	10	Don't know
Specific	○	○	○	○	○	○	○	○	○	○	○	○
Ambitious	○	○	○	○	○	○	○	○	○	○	○	○
Measurable	○	○	○	○	○	○	○	○	○	○	○	○
Realistic	○	○	○	○	○	○	○	○	○	○	○	○
Unambiguous	○	○	○	○	○	○	○	○	○	○	○	○
Scalable	○	○	○	○	○	○	○	○	○	○	○	○

Element 13.2:

"By 2020, the genetic diversity of [. . .] **farmed and domesticated animals** [. . .] is maintained"

	0	1	2	3	4	5	6	7	8	9	10	Don't know
Specific	○	○	○	○	○	○	○	○	○	○	○	○
Ambitious	○	○	○	○	○	○	○	○	○	○	○	○
Measurable	○	○	○	○	○	○	○	○	○	○	○	○
Realistic	○	○	○	○	○	○	○	○	○	○	○	○
Unambiguous	○	○	○	○	○	○	○	○	○	○	○	○
Scalable	○	○	○	○	○	○	○	○	○	○	○	○

Element 13.3:

"By 2020, the genetic diversity of [. . .] **wild relatives** [. . .] is maintained"

	0	1	2	3	4	5	6	7	8	9	10	Don't know
Specific	○	○	○	○	○	○	○	○	○	○	○	○
Ambitious	○	○	○	○	○	○	○	○	○	○	○	○
Measurable	○	○	○	○	○	○	○	○	○	○	○	○
Realistic	○	○	○	○	○	○	○	○	○	○	○	○
Unambiguous	○	○	○	○	○	○	○	○	○	○	○	○
Scalable	○	○	○	○	○	○	○	○	○	○	○	○

Element 13.4:
"By 2020, the genetic diversity of [. . .] **socioeconomically as well as culturally valuable species**, is maintained"

	0	1	2	3	4	5	6	7	8	9	10	Don't know
Specific	○	○	○	○	○	○	○	○	○	○	○	○
Ambitious	○	○	○	○	○	○	○	○	○	○	○	○
Measurable	○	○	○	○	○	○	○	○	○	○	○	○
Realistic	○	○	○	○	○	○	○	○	○	○	○	○
Unambiguous	○	○	○	○	○	○	○	○	○	○	○	○
Scalable	○	○	○	○	○	○	○	○	○	○	○	○

Element 13.5:
"By 2020, [. . .] strategies have been developed and implemented for minimizing genetic erosion and safeguarding their genetic diversity"

	0	1	2	3	4	5	6	7	8	9	10	Don't know
Specific	○	○	○	○	○	○	○	○	○	○	○	○
Ambitious	○	○	○	○	○	○	○	○	○	○	○	○
Measurable	○	○	○	○	○	○	○	○	○	○	○	○
Realistic	○	○	○	○	○	○	○	○	○	○	○	○
Unambiguous	○	○	○	○	○	○	○	○	○	○	○	○
Scalable	○	○	○	○	○	○	○	○	○	○	○	○

Comments (optional)

Assessing the adequacy and effectiveness of the Aichi Biodiversity Targets

Target 14

Please score the Target/elements presented on this page from 0 to 10 for the criteria listed below. One criterion applies to the overall Target ("Comprehensive"); the others apply to individual elements of the Target.

You must provide a score for every row before being able to move onto the next page. Your answers are saved when you click "Save and Next". To leave the page without saving, click the "Exit" button in the top right-hand corner of the page.

A score of 0 means the Target/element does not fit the criterion at all.
A score of 10 means the Target/element completely fits the criterion.

Criteria

- **Comprehensive: the Target covers all important aspects of the issue that it seeks to address**
- **Specific: the Target element sets out clear and well-defined objectives (e.g. quantified percentages, precisely defined terms etc)**
- **Ambitious: the Target element is ambitious and aims sufficiently high to achieve the overall mission to halt the loss of biodiversity**
- **Measurable: progress towards the Target element can be assessed using data already available or feasible to mobilise by 2020 (e.g. quantitative indicators exist or are realistic to produce by 2020)**
- **Realistic: the Target element can feasibly be achieved considering time-frame, practicalities, plausible funding etc**
- **Unambiguous: the Target element is easy to understand and interpret with a single, clear definition**
- **Scalable: the Target element is applicable at global, regional and national scales**

Scroll down or click "OK" to begin scoring this page

Target 14:
"By 2020, ecosystems that provide essential services, including services related to water, and contribute to health, livelihoods, and well-being, are restored and safeguarded, taking into account the needs of women, indigenous and local communities, and the poor and vulnerable."

	0	1	2	3	4	5	6	7	8	9	10	Don't know
Comprehensive	◯	◯	◯	◯	◯	◯	◯	◯	◯	◯	◯	◯

Element 14.1:
"By 2020, ecosystems that provide essential services, including services related to water, and contributing to health, livelihoods, and wellbeing, are restored and safeguarded"

	0	1	2	3	4	5	6	7	8	9	10	Don't know
Specific	◯	◯	◯	◯	◯	◯	◯	◯	◯	◯	◯	◯
Ambitious	◯	◯	◯	◯	◯	◯	◯	◯	◯	◯	◯	◯
Measurable	◯	◯	◯	◯	◯	◯	◯	◯	◯	◯	◯	◯
Realistic	◯	◯	◯	◯	◯	◯	◯	◯	◯	◯	◯	◯
Unambiguous	◯	◯	◯	◯	◯	◯	◯	◯	◯	◯	◯	◯
Scalable	◯	◯	◯	◯	◯	◯	◯	◯	◯	◯	◯	◯

Element 14.2:
"By 2020, [. . . ecosystems are restored and safeguarded] taking into account the needs of women, indigenous and local communities, and the poor and vulnerable"

	0	1	2	3	4	5	6	7	8	9	10	Don't know
Specific												
Ambitious												
Measurable												
Realistic												
Unambiguous												
Scalable												

Comments (optional)

Assessing the adequacy and effectiveness of the Aichi Biodiversity Targets

Target 15

Please score the Target/elements presented on this page from 0 to 10 for the criteria listed below. One criterion applies to the overall Target ("Comprehensive"); the others apply to individual elements of the Target.

You must provide a score for every row before being able to move onto the next page. Your answers are saved when you click "Save and Next". To leave the page without saving, click the "Exit" button in the top right-hand corner of the page.

A score of 0 means the Target/element does not fit the criterion at all.
A score of 10 means the Target/element completely fits the criterion.

<u>Criteria</u>

- **Comprehensive: the Target covers all important aspects of the issue that it seeks to address**
- **Specific: the Target element sets out clear and well-defined objectives (e.g. quantified percentages, precisely defined terms etc)**
- **Ambitious: the Target element is ambitious and aims sufficiently high to achieve the overall mission to halt the loss of biodiversity**
- **Measurable: progress towards the Target element can be assessed using data already available or feasible to mobilise by 2020 (e.g. quantitative indicators exist or are realistic to produce by 2020)**

- **Realistic: the Target element can feasibly be achieved considering time-frame, practicalities, plausible funding etc**
- **Unambiguous: the Target element is easy to understand and interpret with a single, clear definition**
- **Scalable: the Target element is applicable at global, regional and national scales**

Scroll down or click "OK" to begin scoring this page

Target 15:
"By 2020, ecosystem resilience and the contribution of biodiversity to carbon stocks has been enhanced, through conservation and restoration, including restoration of at least 15% of degraded ecosystems, thereby contributing to climate change mitigation and adaptation and to combating desertification."

	0	1	2	3	4	5	6	7	8	9	10	Don't know
Comprehensive	○	○	○	○	○	○	○	○	○	○	○	○

Element 15.1:
"By 2020, ecosystem resilience and the contribution of biodiversity to carbon stocks has been enhanced, through conservation and restoration [. . .] thereby contributing to climate change mitigation and adaptation and to combating desertification"

	0	1	2	3	4	5	6	7	8	9	10	Don't know
Specific	○	○	○	○	○	○	○	○	○	○	○	○
Ambitious	○	○	○	○	○	○	○	○	○	○	○	○
Measurable	○	○	○	○	○	○	○	○	○	○	○	○
Realistic	○	○	○	○	○	○	○	○	○	○	○	○
Unambiguous	○	○	○	○	○	○	○	○	○	○	○	○
Scalable	○	○	○	○	○	○	○	○	○	○	○	○

Element 15.2:
"[. . .] including restoration of at least 15% of degraded ecosystems [by 2020]"

	0	1	2	3	4	5	6	7	8	9	10	Don't know
Specific	○	○	○	○	○	○	○	○	○	○	○	○
Ambitious	○	○	○	○	○	○	○	○	○	○	○	○
Measurable	○	○	○	○	○	○	○	○	○	○	○	○
Realistic	○	○	○	○	○	○	○	○	○	○	○	○
Unambiguous	○	○	○	○	○	○	○	○	○	○	○	○
Scalable	○	○	○	○	○	○	○	○	○	○	○	○

Comments (optional)

Assessing the adequacy and effectiveness of the Aichi Biodiversity Targets

Target 16

Please score the Target/elements presented on this page from 0 to 10 for the criteria listed below. One criterion applies to the overall Target ("Comprehensive"); the others apply to individual elements of the Target.

You must provide a score for every row before being able to move onto the next page. Your answers are saved when you click "Save and Next". To leave the page without saving, click the "Exit" button in the top right-hand corner of the page.

A score of 0 means the Target/element does not fit the criterion at all.
A score of 10 means the Target/element completely fits the criterion.

Criteria

- **Comprehensive: the Target covers all important aspects of the issue that it seeks to address**
- **Specific: the Target element sets out clear and well-defined objectives (e.g. quantified percentages, precisely defined terms etc)**
- **Ambitious: the Target element is ambitious and aims sufficiently high to achieve the overall mission to halt the loss of biodiversity**
- **Measurable: progress towards the Target element can be assessed using data already available or feasible to mobilise by 2020 (e.g. quantitative indicators exist or are realistic to produce by 2020)**
- **Realistic: the Target element can feasibly be achieved considering time-frame, practicalities, plausible funding etc**
- **Unambiguous: the Target element is easy to understand and interpret with a single, clear definition**
- **Scalable: the Target element is applicable at global, regional and national scales**

Scroll down or click "OK" to begin scoring this page

Target 16:
"By 2015, the Nagoya Protocol on Access to Genetic Resources and the Fair and Equitable Sharing of Benefits Arising from their Utilization is in force and operational, consistent with national legislation."

	0	1	2	3	4	5	6	7	8	9	10	Don't know
Comprehensive	○	○	○	○	○	○	○	○	○	○	○	○

Element 16.1:
"By 2015, the Nagoya Protocol on Access to Genetic Resources and the Fair and Equitable Sharing of Benefits Arising from their Utilization **is in force**"

	0	1	2	3	4	5	6	7	8	9	10	Don't know
Specific												
Ambitious												
Measurable												
Realistic												
Unambiguous												
Scalable												

Element 16.2:
"By 2015, the Nagoya Protocol on Access to Genetic Resources and the Fair and Equitable Sharing of Benefits Arising from their Utilization [. . .] **is operational, consistent with national legislation**"

	0	1	2	3	4	5	6	7	8	9	10	Don't know
Specific												
Ambitious												
Measurable												
Realistic												
Unambiguous												
Scalable												

Comments (optional)

Assessing the adequacy and effectiveness of the Aichi Biodiversity Targets

Target 17

Please score the Target/elements presented on this page from 0 to 10 for the criteria listed below. One criterion applies to the overall Target ("Comprehensive"); the others apply to individual elements of the Target.

You must provide a score for every row before being able to move onto the next page. Your answers are saved when you click "Save and Next". To leave the page without saving, click the "Exit" button in the top right-hand corner of the page.

A score of 0 means the Target/element does not fit the criterion at all.
A score of 10 means the Target/element completely fits the criterion.

Criteria

- **Comprehensive: the Target covers all important aspects of the issue that it seeks to address**
- **Specific: the Target element sets out clear and well-defined objectives (e.g. quantified percentages, precisely defined terms etc)**
- **Ambitious: the Target element is ambitious and aims sufficiently high to achieve the overall mission to halt the loss of biodiversity**
- **Measurable: progress towards the Target element can be assessed using data already available or feasible to mobilise by 2020 (e.g. quantitative indicators exist or are realistic to produce by 2020)**
- **Realistic: the Target element can feasibly be achieved considering time-frame, practicalities, plausible funding etc**
- **Unambiguous: the Target element is easy to understand and interpret with a single, clear definition**
- **Scalable: the Target element is applicable at global, regional and national scales**

Scroll down or click "OK" to begin scoring this page

Target 17:
"By 2015, each party has developed, adopted as a policy instrument, and has commenced implementing an effective, participatory and updated national biodiversity strategy and action plan."

	0	1	2	3	4	5	6	7	8	9	10	Don't know
Comprehensive	○	○	○	○	○	○	○	○	○	○	○	○

Element 17.1:
"By 2015, each Party **has developed** [. . .] an effective, participatory, and updated national biodiversity strategy and action plan"

	0	1	2	3	4	5	6	7	8	9	10	Don't know
Specific	○	○	○	○	○	○	○	○	○	○	○	○
Ambitious	○	○	○	○	○	○	○	○	○	○	○	○
Measurable	○	○	○	○	○	○	○	○	○	○	○	○
Realistic	○	○	○	○	○	○	○	○	○	○	○	○
Unambiguous	○	○	○	○	○	○	○	○	○	○	○	○
Scalable	○	○	○	○	○	○	○	○	○	○	○	○

Element 17.2:

"By 2015, each party **has** [. . .]**adopted as a policy instrument** [. . .] an effective, participatory, and updated national biodiversity strategy and action plan"

	0	1	2	3	4	5	6	7	8	9	10	Don't know
Specific												
Ambitious												
Measurable												
Realistic												
Unambiguous												
Scalable												

Element 17.3

"By 2015, each party [. . .]**has commenced implementing** an effective, participatory, and updated national biodiversity strategy and action plan"

	0	1	2	3	4	5	6	7	8	9	10	Don't know
Specific												
Ambitious												
Measurable												
Realistic												
Unambiguous												
Scalable												

Comments (optional)

Assessing the adequacy and effectiveness of the Aichi Biodiversity Targets

Target 18

Please score the Target/elements presented on this page from 0 to 10 for the criteria listed below. One criterion applies to the overall Target ("Comprehensive"); the others apply to individual elements of the Target.

You must provide a score for every row before being able to move onto the next page. Your answers are saved when you click "Save and Next". To leave the page without saving, click the "Exit" button in the top right-hand corner of the page.

A score of 0 means the Target/element does not fit the criterion at all.
A score of 10 means the Target/element completely fits the criterion.

Criteria

- **Comprehensive: the Target covers all important aspects of the issue that it seeks to address**
- **Specific: the Target element sets out clear and well-defined objectives (e.g. quantified percentages, precisely defined terms etc)**
- **Ambitious: the Target element is ambitious and aims sufficiently high to achieve the overall mission to halt the loss of biodiversity**
- **Measurable: progress towards the Target element can be assessed using data already available or feasible to mobilise by 2020 (e.g. quantitative indicators exist or are realistic to produce by 2020)**
- **Realistic: the Target element can feasibly be achieved considering time-frame, practicalities, plausible funding etc**
- **Unambiguous: the Target element is easy to understand and interpret with a single, clear definition**
- **Scalable: the Target element is applicable at global, regional and national scales**

Scroll down or click "OK" to begin scoring this page

Target 18:
"By 2020, the traditional knowledge, innovation, and practices of indigenous and local communities relevant for the conservation and sustainable use of biodiversity, and their customary use of biological resources, are respected, subject to national legislation and relevant international obligations, and fully integrated and reflected in the implementation of the convention with the full and effective participation of indigenous and local communities, at all relevant levels."

	0	1	2	3	4	5	6	7	8	9	10	Don't know
Comprehensive	○	○	○	○	○	○	○	○	○	○	○	○

Element 18.1:
"By 2020, the traditional knowledge, innovations and practices of indigenous and local communities relevant for the conservation and sustainable use of biodiversity, and their customary use of biological resources, **are respected, subject to national legislation and relevant international obligations**[. . .] at all relevant levels."

	0	1	2	3	4	5	6	7	8	9	10	Don't know
Specific	○	○	○	○	○	○	○	○	○	○	○	○
Ambitious	○	○	○	○	○	○	○	○	○	○	○	○
Measurable	○	○	○	○	○	○	○	○	○	○	○	○
Realistic	○	○	○	○	○	○	○	○	○	○	○	○
Unambiguous	○	○	○	○	○	○	○	○	○	○	○	○
Scalable	○	○	○	○	○	○	○	○	○	○	○	○

Element 18.2:
"By 2020, the traditional knowledge, innovations, and practices of indigenous and local communities relevant for the conservation and sustainable use of biodiversity, and their customary use of biological resources, **are** [. . .]**fully integrated and reflected in the implementation of the convention**[. . .] at all relevant levels."

	0	1	2	3	4	5	6	7	8	9	10	Don't know
Specific	○	○	○	○	○	○	○	○	○	○	○	○
Ambitious	○	○	○	○	○	○	○	○	○	○	○	○
Measurable	○	○	○	○	○	○	○	○	○	○	○	○
Realistic	○	○	○	○	○	○	○	○	○	○	○	○
Unambiguous	○	○	○	○	○	○	○	○	○	○	○	○
Scalable	○	○	○	○	○	○	○	○	○	○	○	○

Element 18.3
"By 2020, the traditional knowledge, innovations, and practices of indigenous and local communities relevant for the conservation and sustainable use of biodiversity, and their customary use of biological resources, [are respected, integrated, and reflected] **with the full and effective participation of indigenous and local communities**, at all relevant levels."

	0	1	2	3	4	5	6	7	8	9	10	Don't know
Specific	○	○	○	○	○	○	○	○	○	○	○	○
Ambitious	○	○	○	○	○	○	○	○	○	○	○	○
Measurable	○	○	○	○	○	○	○	○	○	○	○	○
Realistic	○	○	○	○	○	○	○	○	○	○	○	○
Unambiguous	○	○	○	○	○	○	○	○	○	○	○	○
Scalable	○	○	○	○	○	○	○	○	○	○	○	○

Comments (optional)

Target 19

Please score the Target/elements presented on this page from 0 to 10 for the criteria listed below. One criterion applies to the overall Target ("Comprehensive"); the others apply to individual elements of the Target.

You must provide a score for every row before being able to move onto the next page. Your answers are saved when you click "Save and Next". To leave the page without saving, click the "Exit" button in the top right-hand corner of the page.

A score of 0 means the Target/element does not fit the criterion at all.
A score of 10 means the Target/element completely fits the criterion.

<u>**Criteria**</u>

- **Comprehensive: the Target covers all important aspects of the issue that it seeks to address**
- **Specific: the Target element sets out clear and well-defined objectives (e.g. quantified percentages, precisely defined terms etc)**
- **Ambitious: the Target element is ambitious and aims sufficiently high to achieve the overall mission to halt the loss of biodiversity**
- **Measurable: progress towards the Target element can be assessed using data already available or feasible to mobilise by 2020 (e.g. quantitative indicators exist or are realistic to produce by 2020)**
- **Realistic: the Target element can feasibly be achieved considering time-frame, practicalities, plausible funding etc**
- **Unambiguous: the Target element is easy to understand and interpret with a single, clear definition**
- **Scalable: the Target element is applicable at global, regional and national scales**

Scroll down or click "OK" to begin scoring this page

Target 19:
"By 2020, knowledge, the science base, and technologies relating to biodiversity, its values, functioning, status and trends, and the consequences of its loss, are improved, widely shared and transferred, and applied."

	0	1	2	3	4	5	6	7	8	9	10	Don't know
Comprehensive	○	○	○	○	○	○	○	○	○	○	○	○

Element 19.1:
"By 2020, knowledge, the science base and technologies relating to biodiversity, its values, functioning, status and trends, and the consequences of its loss, **are improved, widely shared, and transferred**[. . .]"

	0	1	2	3	4	5	6	7	8	9	10	Don't know
Specific	○	○	○	○	○	○	○	○	○	○	○	○
Ambitious	○	○	○	○	○	○	○	○	○	○	○	○
Measurable	○	○	○	○	○	○	○	○	○	○	○	○
Realistic	○	○	○	○	○	○	○	○	○	○	○	○
Unambiguous	○	○	○	○	○	○	○	○	○	○	○	○
Scalable	○	○	○	○	○	○	○	○	○	○	○	○

Element 19.2:
"By 2020, knowledge, the science base and technologies relating to biodiversity, its values, functioning, status and trends, and the consequences of its loss, **are** [. . .]**applied**."

	0	1	2	3	4	5	6	7	8	9	10	Don't know
Specific	○	○	○	○	○	○	○	○	○	○	○	○
Ambitious	○	○	○	○	○	○	○	○	○	○	○	○
Measurable	○	○	○	○	○	○	○	○	○	○	○	○
Realistic	○	○	○	○	○	○	○	○	○	○	○	○
Unambiguous	○	○	○	○	○	○	○	○	○	○	○	○
Scalable	○	○	○	○	○	○	○	○	○	○	○	○

Comments (optional)

Assessing the adequacy and effectiveness of the Aichi Biodiversity Targets

Target 20

Please score the Target/elements presented on this page from 0 to 10 for the criteria listed below. One criterion applies to the overall Target ("Comprehensive"); the others apply to individual elements of the Target.

You must provide a score for every row before being able to move onto the next page. Your answers are saved when you click "Save and Next". To leave the page without saving, click the "Exit" button in the top right-hand corner of the page.

A score of 0 means the Target/element does not fit the criterion at all.
A score of 10 means the Target/element completely fits the criterion.

Criteria

- **Comprehensive: the Target covers all important aspects of the issue that it seeks to address**
- **Specific: the Target element sets out clear and well-defined objectives (e.g. quantified percentages, precisely defined terms etc)**
- **Ambitious: the Target element is ambitious and aims sufficiently high to achieve the overall mission to halt the loss of biodiversity**
- **Measurable: progress towards the Target element can be assessed using data already available or feasible to mobilise by 2020 (e.g. quantitative indicators exist or are realistic to produce by 2020)**

- **Realistic: the Target element can feasibly be achieved considering time-frame, practicalities, plausible funding etc**
- **Unambiguous: the Target element is easy to understand and interpret with a single, clear definition**
- **Scalable: the Target element is applicable at global, regional and national scales**

Scroll down or click "OK" to begin scoring this page

Target 20:
"By 2020, at the latest, the mobilization of financial resources for effectively implementing the Strategic Plan for Biodiversity 2011–2020 from all sources, and in accordance with the consolidated and agreed process in the Strategy for Resource Mobilization, should increase substantially from the current levels. This target will be subject to changes contingent to resource needs, assessments to be developed and reported by Parties."

	0	1	2	3	4	5	6	7	8	9	10	Don't know
Comprehensive	○	○	○	○	○	○	○	○	○	○	○	○

Element 20.1:
"By 2020, [. . .] the mobilization of financial resources for effectively implementing the Strategic Plan for Biodiversity 2011–2020 from all sources, and in accordance with the consolidated and agreed process in the Strategy for Resource Mobilization, should increase substantially from the current levels [. . .]"

	0	1	2	3	4	5	6	7	8	9	10	Don't know
Specific	○	○	○	○	○	○	○	○	○	○	○	○
Ambitious	○	○	○	○	○	○	○	○	○	○	○	○
Measurable	○	○	○	○	○	○	○	○	○	○	○	○
Realistic	○	○	○	○	○	○	○	○	○	○	○	○
Unambiguous	○	○	○	○	○	○	○	○	○	○	○	○
Scalable	○	○	○	○	○	○	○	○	○	○	○	○

Comments (optional)

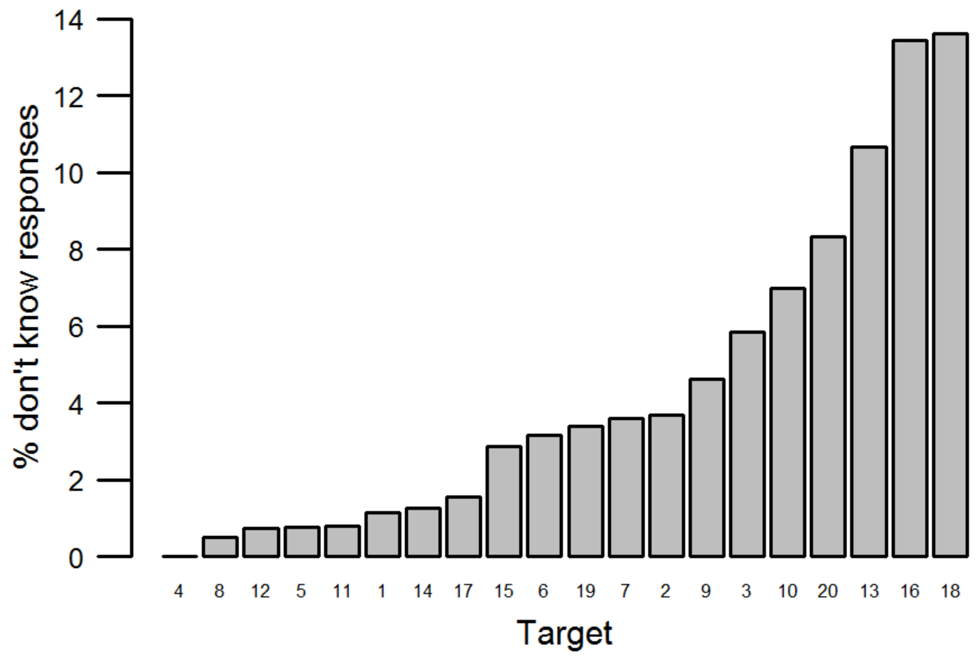

Appendix 12

Percentage of scores per Target (calculated across all criteria and all constituent elements) that were "Don't know".

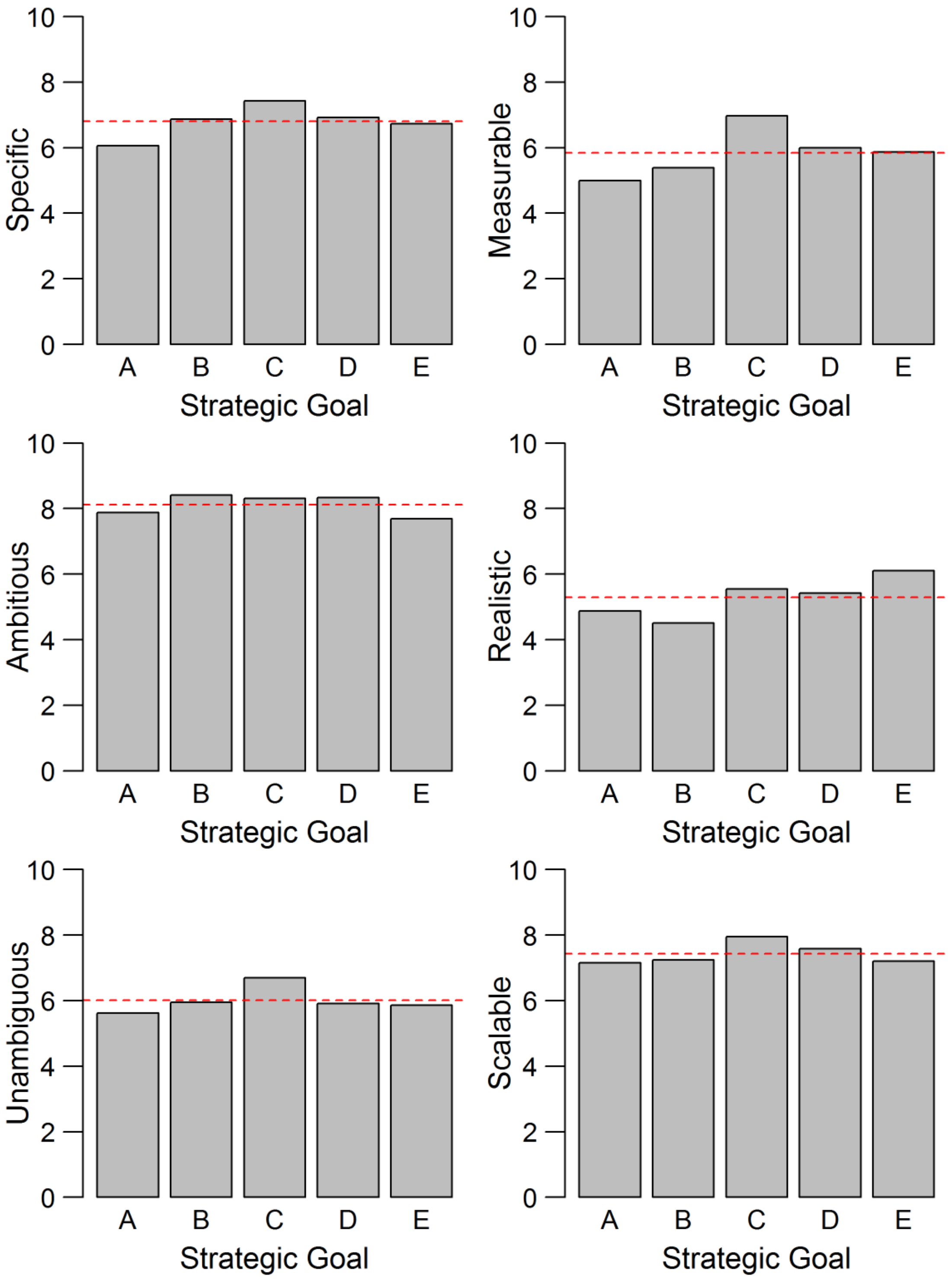

Appendix 13

SMART scores per Strategic Goal, calculated by averaging across scores per Target per Strategic Goal for each element-level criterion. The dashed red line indicates the mean score across all Strategic Goals.
